# Retinal Capillary Density Reduction Contributes to Dysthyroid Optic Neuropathy via an L‐Arginine‐NO Pathway: A Metabonomics and Clinical Trial Study

**DOI:** 10.1002/mco2.70652

**Published:** 2026-03-04

**Authors:** Yunhai Tu, Congcong Yan, Lu Chen, Weijie Liu, Xiaozhou Hu, Mengyuan Gao, Wei Rao, Jiayi Zhang, Junye Zhu, Hui Wu, Kang Zhang, Meng Zhou, Wencan Wu

**Affiliations:** ^1^ State Key Laboratory of Ophthalmology Optometry and Visual Science Eye Hospital Wenzhou Medical University Wenzhou China; ^2^ National Clinical Research Center for Ocular Diseases Eye Hospital Wenzhou Medical University Wenzhou China; ^3^ School of Biomedical Engineering Eye Hospital Wenzhou Medical University Wenzhou China; ^4^ Institute For Advanced Study On Eye Disease and Health Wenzhou Medical University Wenzhou Zhejiang China; ^5^ Zhejiang Key Laboratory of Core Technologies For Reconstruction of Ocular‐Cerebral Neural Pathway Functions Zhejiang China; ^6^ Oujiang Laboratory (Zhejiang Lab For Regenerative Medicine Vision and Brain Health) Zhejiang China

**Keywords:** dysthyroid optic neuropathy, L‐arginine, nitric oxide, retinal capillary density, thyroid‐associated ophthalmopathy

## Abstract

Dysthyroid optic neuropathy (DON) is the most severe complication of thyroid‐associated ophthalmopathy (TAO). Although recent evidence indicates that reduced retinal capillary density (RCD) may increase DON risk independently of orbital apex crowding, the underlying mechanisms and associated metabolic reprogramming remain unclear. In a retrospective analysis of TAO patients with and without DON, those with DON demonstrated elevated pulse pressure (PP), decreased RCD, and higher incidences of dyslipidemia, hyperglycemia, and internal carotid artery calcification. To explore the metabolic basis of these findings, untargeted and targeted metabolomic profiling of plasma from TAO patients and healthy controls was conducted, identifying DON‐associated abnormalities in the L‐arginine metabolic pathway (registration number: ChiCTR2000035598). Integrating these results with existing literature suggests that oxidative stress drives dysregulated L‐arginine–nitric oxide (NO) metabolism, contributing to progressive RCD loss. In early‐stage DON patients treated with oral L‐arginine, improvements in RCD, PP, and visual function were observed (registration number: ChiCTR2300076962). Further analyses implicated reduced NO bioavailability, due to L‐arginine depletion and endothelial NO synthase (eNOS) uncoupling, as a key contributor to declining RCD. Given that oral L‐arginine can improve PP via NO‐mediated pathways in cardiovascular disease, our findings offer a promising new therapeutic direction for DON management.

## Graphical abstract

Oxidative stress induces endothelial nitric oxide synthase (eNOS) uncoupling and L‐arginine depletion, reducing nitric oxide (NO) bioavailability in vascular endothelium and contributing to decreased retinal capillary density (RCD) in dysthyroid optic neuropathy (DON). This mechanism also explains the elevated pulse pressure (PP), increased incidence of arteriosclerosis, and systemic risk factors such as advanced age, hyperlipidemia, and smoking in DON.

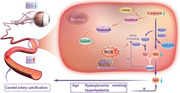

## 1 Introduction

Thyroid‐associated ophthalmopathy (TAO), also known as Graves’ orbitopathy (GO), is an orbital disorder characterized by thyroid dysfunction and extensive remodeling of orbital tissues [1, 2]. Clinical manifestations range from eyelid retraction and proptosis to sight‐threatening dysthyroid optic neuropathy (DON), which occurs in approximately 3%–14.3% of TAO patients and can cause irreversible blindness if not promptly treated [3, 4]. Current therapeutic options for DON, including intravenous glucocorticoids, orbital decompression surgery, and emerging targeted agents, remain limited by substantial side effects, variable efficacy, and high rates of recurrence, primarily reflecting gaps in our understanding of disease pathogenesis [5].

Although DON has traditionally been attributed primarily to mechanical compression [6], our previous work demonstrated that DON patients show abnormal blue–yellow color vision and impaired contrast sensitivity even in the absence of direct optic nerve compression or orbital apex crowding [7, 8]. Several recognized DON risk factors, including older age, smoking, diabetes, and hyperlipidemia, are likewise unrelated to local mechanical forces, suggesting the presence of additional pathogenic mechanisms [9, 10, 11].

A growing body of evidence implicates decreased retinal capillary density (RCD) as both an early biomarker and functional mediator of DON, given its strong association with visual field deficits [12, 13, 14, 15]. RCD continues to decline even after orbital decompression surgery [16, 17], indicating that mechanical factors alone cannot fully explain this phenomenon. Consistent with this, our cohort of early‐stage DON patients without orbital apex crowding demonstrated significant correlations between RCD and quick contrast sensitivity function (qCSF) performance [8]. Elevated pulse pressure (PP), a marker of impaired arterial elasticity, has also been identified as an independent risk factor for reduced RCD [18], further suggesting systemic vascular abnormalities. This aligns with single‐cell sequencing studies identifying endothelial cells, key regulators of vascular homeostasis, as central contributors to TAO pathogenesis [19].

These observations led us to hypothesize that the mechanisms driving RCD reduction in DON overlap with those underlying systemic vasculopathies, particularly through shared endothelial dysfunction. Endothelial cells are essential for maintaining capillary integrity and vasodilatory capacity, processes that depend on tightly regulated metabolic pathways, including glycolysis [20], fatty acid oxidation [21], and amino acid metabolism [22]. Disruption of these pathways can lead to endothelial dysfunction, a hallmark of vascular disease [23], reflected by characteristic changes in circulating metabolites.

To clarify the relationship between systemic vasculopathy and RCD reduction in DON, this study pursued three objectives: (1) to establish clinical evidence of systemic vascular impairment in DON patients; (2) to identify specific metabolic abnormalities associated with this vascular dysfunction; and (3) to evaluate the protective effects of L‐arginine supplementation on RCD and visual function, as assessed by qCSF. Through integrated clinical and metabolomic analyses, this study aimed to elucidate the metabolic mechanisms linking systemic vasculopathy to RCD decline and to identify potential therapeutic targets for DON.

## 2 Results

### 2.1 Retrospective Analyses Suggest That Large Vascular Abnormalities May Be Related to RCD in DON Patients

The retrospective clinical analysis included 357 patients, comprising 164 with DON and 193 without DON (Tables 1 and  2). Baseline comparisons between the two groups revealed that patients with DON had significantly higher PP than those without DON (*p* = 0.004; Table 1). DON was also associated with elevated levels of glycated hemoglobin (HbA1c), fasting plasma glucose (FPG), triglycerides (TG), and the triglyceride–glucose (TyG) index. Similarly, the incidence of internal carotid artery calcification (ICAC) was significantly higher among DON patients (*p* < 0.001, Table 1). To further investigate the link between macrovascular and microvascular changes, the correlation between PP and macular RCD was assessed. PP was significantly negatively correlated with RCD, indicating that higher PP is associated with greater reductions in RCD (Figure S1).

**TABLE 1:** A retrospective cohort study reveals different characteristics between TAO and DON patients.

Characteristic	Non‐DON (*N* = 193)	DON (*N* = 164)	*p*‐value
Eye (*n*)	375	228	
Age (years)	**47.0** (**18.0**)	**55.5** (**17.0**)	**<0.001** ^a^
Gender (*n*)			0.525^b^
Female	101	80	
Male	92	84	
BMI (kg/m^2^)	**23.6** (**4.4**)	**24.9** (**4.1**)	**0.004** ^a^
SBP (mmHg)	**126.0** (**30**)	**129.5** (**26**)	**0.013** ^a^
DBP (mmHg)	80.0 (17)	83.0 (13)	0.289^a^
PP (mmHg)	**44.0** (**17**)	**48.0** (**21**)	**0.004** ^a^
Hypertension (*n*)			0.087^b^
No	115	82	
Yes	78	82	
Diabetes mellitus (*n*)			**0.021** ^b^
No	170	129	
Yes	23	35	
Hyperlipidemia (*n*)			0.439^b^
No	128	102	
Yes	65	62	
HbA1c (%)	**5.60** (**0.60**)	**5.80** (**0.70**)	**<0.001** ^a^
FPG (mmol/L)	**5.30** (**0.82**)	**5.47** (**0.84**)	**0.011** ^a^
LDL (mmol/L)	2.99 (1.16)	3.00 (1.13)	0.765^a^
HDL (mmol/L)	1.33 (0.51)	1.36 (0.46)	0.769^a^
TC (mmol/L)	4.94 (1.55)	5.00 (1.30)	0.434^a^
TG (mmol/L)	**1.24** (**1.09**)	**1.38** (**1.14**)	**0.039** ^a^
TyG	**6.95** (**0.87**)	**7.16** (**0.83**)	**0.012** ^a^
ICAC (*n*, %)^c^	**234** (**62.4**)	**178** (**78.1**)	**<0.001** ^b^

*Note*: Data are presented as median (interquartile range) or number (percentage). Bold vaules indicate statistically significant differences between the Non‐DON and DON groups (*p*<0.05).

Abbreviations: BMI, body mass index; DBP, diastolic blood pressure; DON, dysthyroid optic neuropathy; FPG, fasting plasma glucose; HbA1c, glycated hemoglobin; HDL, high‐density lipoprotein; ICAC, internal carotid artery calcification; LDL, low‐density lipoprotein; Non‐DON, TAO patients without DON; PP, pulse pressure; SBP, systolic blood pressure; TC, total cholesterol; TG, triglyceride; TyG, triglyceride‐glucose index.

^a^Mann–Whitney *U* test.

^b^ *χ* ^2^ test.

^c^ICAC was defined as the presence of calcification in at least one internal carotid artery. The percentage represents the proportion of patients with ICAC in each group.

**TABLE 2:** Baseline demographic and clinical characteristics of participants of the study cohorts.

	Cohort 1^a^	Cohort 2^b^						Cohort 3^c^
		Un‐targeted metabolomic analysis			Targeted metabolomic analysis			
Parameters	TAO (*n* = 357)	HC (*n* = 35)	TAO (*n* = 82)	*p*	HC (*n* = 44)	TAO (*n* = 59)	*p*	TAO (*n* = 16)
Basic characteristics								
Age (years)	50.21 ± 12.96	46.60 ± 11.60	47.32 ± 12.70	0.784	48.82 ± 12.70	45.38 ± 13.01	0.187	48.75 ± 8.88
Male, *n* (%)	176 (49.3%)	6 (17.1%)	29 (35.3%)	0.049	9 (25.7%)	29 (49.2%)	0.005	4 (25%)
BMI (kg/m^2^)	24.59 ± 3.53	23.49 ± 3.35	24.75 ± 3.29	0.094	23.14 ± 3.48	24.46 ± 4.66	0.288	
Disease duration (years)	2.53 ± 4.30	NA	2.46 ± 3.57		NA	3.88 ± 5.24		2.14 ± 1.50
CAS								
OD		NA	1.45 ± 1.29		NA	1.36 ± 1.17		0.69 ± 0.79
OS		NA	1.43 ± 1.24		NA	1.43 ± 1.13		0.63 ± 0.89
BCVA (logMAR)								
OD	0.29 ± 0.51		0.02 ± 0.66			0.24 ± 0.43		0.04 ± 0.07
OS	0.31 ± 0.55		0.02 ± 0.80			0.20 ± 0.64		0.06 ± 0.09
Proptosis (mm)								
OD		NA	19.19 ± 3.24		NA	19.34 ± 3.08		16.94 ± 1.88
OS		NA	19.92 ± 3.39		NA	20.38 ± 3.65		17.38 ± 2.09
Thyroid function								
TRAb (IU/L)			7.34 ± 7.74			6.25 ± 9.71		
TSH (mIU/L)	3.68 ± 7.37		5.14 ± 11.28			3.90 ± 10.26		
FT3 (pmol/L)	5.60 ± 3.22		5.68 ± 3.24			5.62 ± 2.86		
FT4 (pmol/L)	17.64 ± 6.97		16.43 ± 5.76			17.47 ± 6.71		
T3 (nmol/L)	1.90 ± 0.80		5.88 ± 21.46			3.52 ± 15.46		
T4 (nmol/L)	103.81 ± 30.55		100.42 ± 34.77			108.78 ± 29.86		
Blood lipids								
TC (mmol/L)	5.01 ± 1.05	4.60 ± 0.72	5.15 ± 1.01	0.009	4.97 ± 1.01	4.96 ± 1.03	0.991	5.33 ± 0.72
TG (mmol/L)	1.70 ± 1.45	1.29 ± 0.95	1.64 ± 2.54	0.431	1.45 ± 1.03	1.64 ± 0.98	0.361	1.80 ± 0.76
LDL (mmol/L)	3.02 ± 0.85	2.59 ± 0.64	3.11 ± 0.83	0.003	2.91 ± 0.85	3.11 ± 0.84	0.244	3.38 ± 0.72
HDL (mmol/L)	1.37 ± 0.36	1.45 ± 0.40	1.39 ± 0.34	0.480	1.44 ± 0.40	3.60 ± 16.84	0.413	1.50 ± 0.27

*Note*: Data are presented as mean ± standard deviation (SD) or number (percentage).

Abbreviations: BCVA, best‐corrected visual acuity; BMI, body mass index; CAS, clinical activity score; DON, dysthyroid optic neuropathy; FT3, free triiodothyronine; FT4, free thyroxine; HC, healthy controls; HDL, high‐density lipoprotein; LDL, low‐density lipoprotein; NA, not applicable; OD: right eye; OS, left eye; T3, total triiodothyronine; T4, total thyroxine; TAO, thyroid‐associated ophthalmopathy; TC, total cholesterol; TG, triglyceride; TRAb, thyrotropin receptor antibody; TSH, thyroid‐stimulating hormone.

^a^ Cohort 1 (retrospective study): Comprised patients with and without DON for the analysis of vascular parameters.

^b^ Cohort 2 (metabolomics study): Included HC and TAO patients for untargeted and targeted metabolomic profiling.

^c^ Cohort 3 (L‐arginine supplementation study): An interventional cohort of patients with early‐stage DON who received oral L‐arginine.

### 2.2 Characterization of TAO‐Related Serum Metabolomic Abnormalities

**FIGURE 1:**
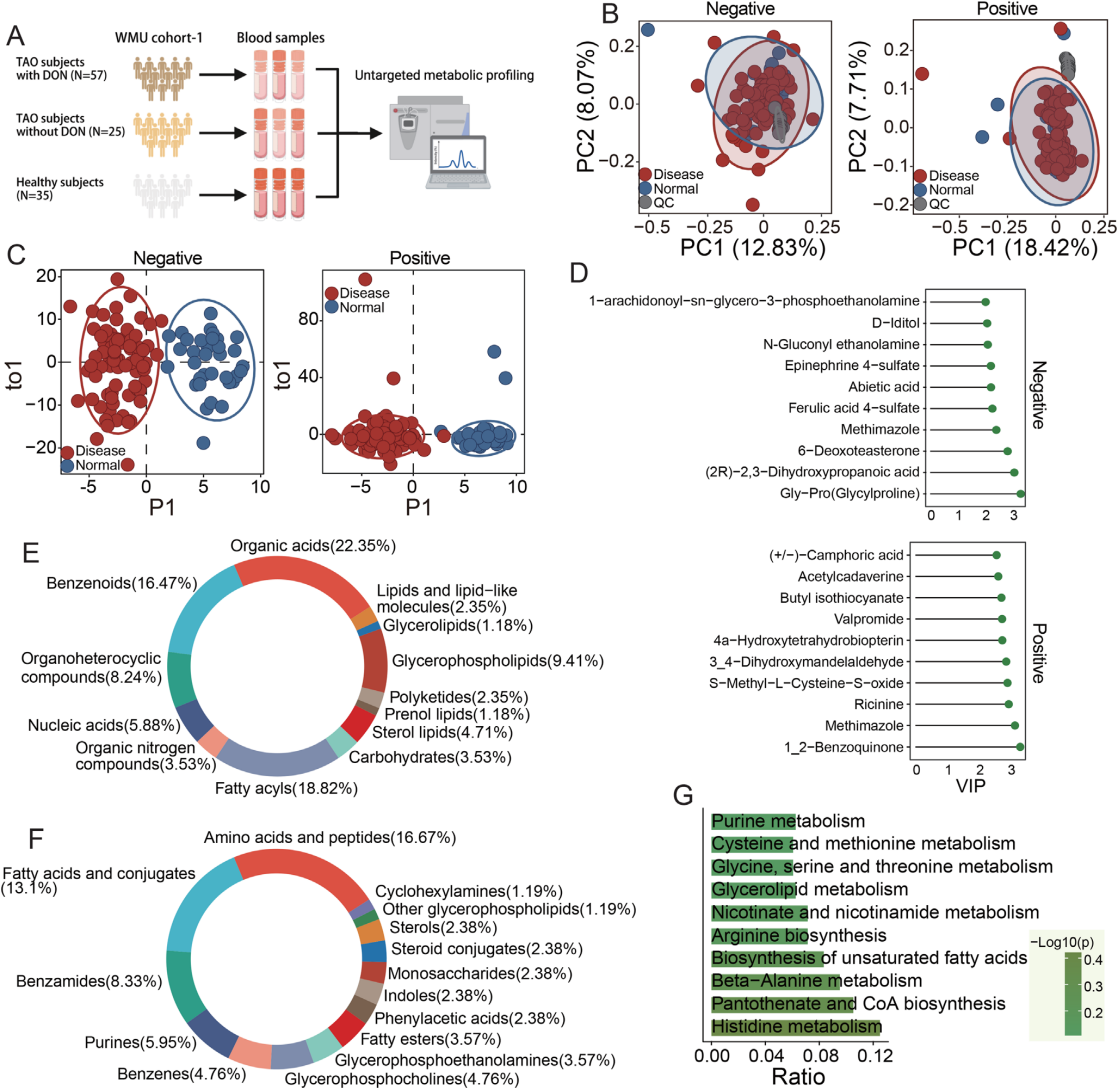
Characterization of dysregulated serum metabolomics in TAO using untargeted metabolomics. (A) Workflow depicting the details of untargeted metabolic profiling of WMU cohort‐1. (B) Principal component analysis (PCA) score plots of metabolic profiles, showing variation in the metabolome among samples. Quality control (QC) samples are shown in gray. (C) Orthogonal partial least squares–discriminant analysis (OPLS‐DA) score plots of TAO patients (*n* = 82) and healthy controls (*n* = 35) of plasma samples based on untargeted metabolomics in ESI‐mode and ESI+ mode. Red: TAO, blue: normal. (D) Top 10 representative significantly differentially expressed metabolites (DEMs) and the variable importance in projection (VIP) values of these metabolites are reported on the horizontal axis. (E and F) Based on the known metabolites, distribution and percentages of the Human Metabolome Database (HMDB) superclass level (E) or class level (F) are summarized in the pie charts. The different colors represent different superclasses or class levels. (G) Bar chart of the top top‐enriched 10 Kyoto Encyclopedia of Genes and Genomes (KEGG) pathways.

Having established an association between macrovascular abnormalities and reduced RCD in DON patients, it was next hypothesized that specific blood‐based metabolic alterations might help explain this connection. To investigate this, untargeted metabolomic profiling of plasma samples using high‐resolution mass spectrometry (Table 2 and Figure 1A) was performed, identifying 12,113 peaks across positive and negative ionization modes. Method reproducibility was assessed by calculating the relative standard deviation (RSD) of peak areas in pooled quality control (QC) samples. In both ESI+ and ESI− modes, 75% of QC peaks demonstrated an RSD below 20%, indicating high data quality (Figure S2A). Only metabolic features with an RSD ≤ 20% were retained for further analyses. Principal component analysis (PCA) demonstrated that QC samples clustered tightly near the origin, confirming minimal technical variability (Figure 1B). The untargeted metabolomics dataset was found to be robust and reliable.

After QC filtering, 9079 metabolites remained for downstream analysis. Supervised orthogonal partial least squares–discriminant analysis (OPLS‐DA) revealed clear separations between TAO patients and healthy controls in both ionization modes, indicating substantial metabolic alterations associated with TAO (Figure 1C). Variable importance in projection (VIP) scores were calculated to assess individual metabolite contributions, and significance was determined using the Mann–Whitney *U* test. In total, 111 (ESI−) and 198 (ESI+) metabolites with VIP ≥ 1 and *p* < 0.05 were identified (Figure S2B and Table S1). Among these, 52 and 159 metabolites were significantly upregulated, and 59 and 39 were downregulated in ESI− and ESI+ modes, respectively, when comparing TAO patients with controls (Table S1). Heatmaps depicting the relative abundance of these differential metabolites are shown in Figure S2C, and the top 10 metabolites are highlighted in Figure 1D.

Differential metabolites were annotated using the Human Metabolome Database (HMDB) and categorized at the class and superclass levels, with their distributions displayed in pie charts (Figure 1E,F). At the superclass level, the most represented groups were organic acids (22.35%), fatty acyls (18.82%), and benzenoids (16.47%). At the class level, amino acids and peptides were most abundant (16.67%), followed by fatty acids and conjugates (13.1%). KEGG pathway enrichment analysis revealed significant enrichment of histidine metabolism and beta‐alanine metabolism, pathways essential for cellular energy production, growth, and survival (Figure 1G and Table S5).

### 2.3 Characterization of DON‐Related Serum Metabolomic Abnormalities

**FIGURE 2:**
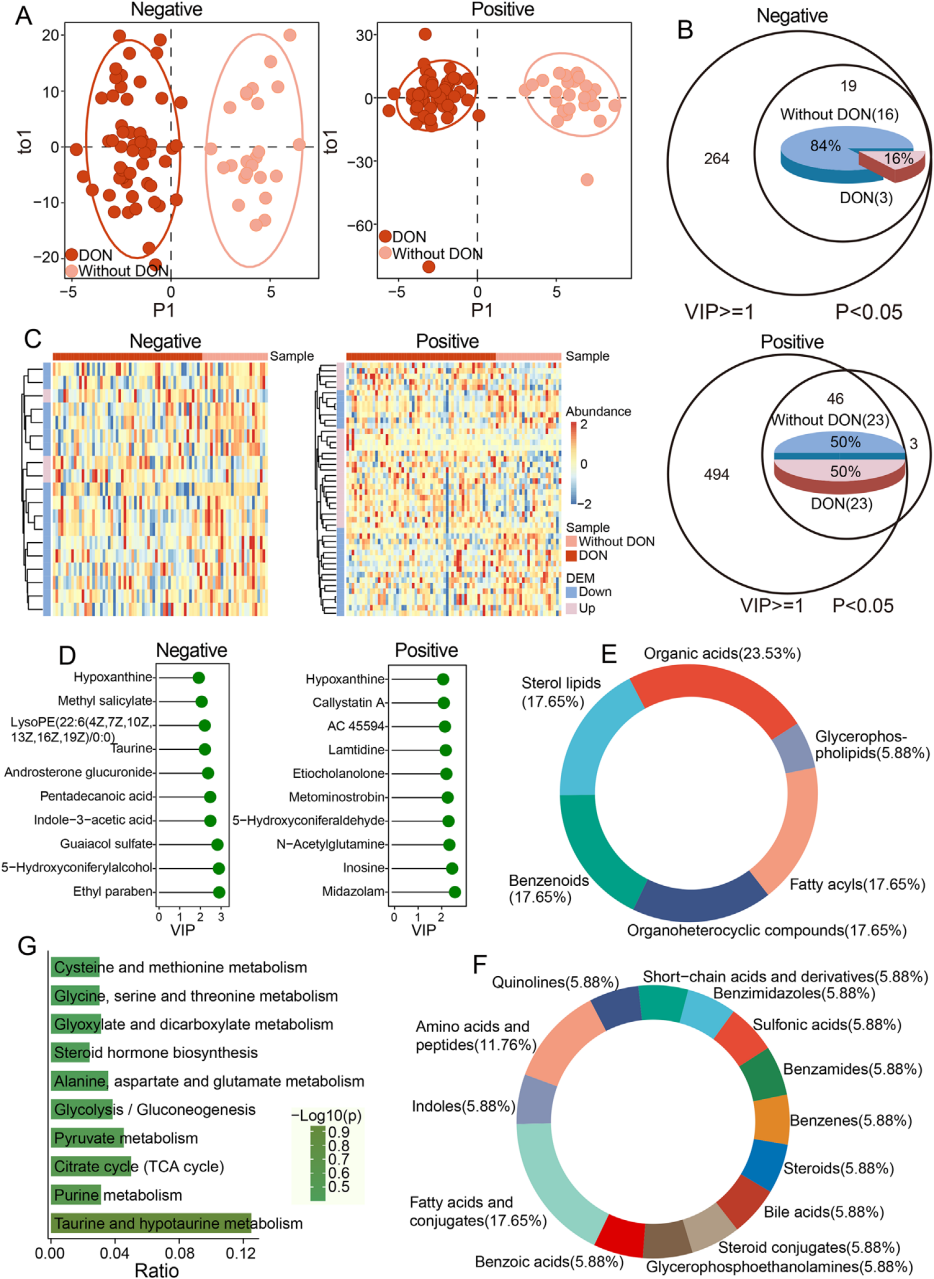
Characterization of dysregulated serum metabolomics in TAO with/without DON using untargeted metabolomics. (A) Plots of orthogonal partial least squares–discriminant analysis (OPLS‐DA) scores for untargeted metabolomics derived from without DON samples (*n* = 25) and with DON samples (*n* = 57) in ESI− mode and ESI+ mode. (B) Venn diagrams demonstrating the overlap between significantly differentially expressed metabolites (DEMs) identified from univariate (*p* < 0.05) and multivariate (VIP ≥ 1) analysis in ESI− mode and ESI+ mode. Red: upregulated in the DON group; blue: upregulated in the group without DON. (C) Heatmap showing the relative abundance of the DEMs. (D) Top 10 representative DEMs. The x‐axis shows the VIP values of metabolites. (E and F) Pie chart showing frequency of the Human Metabolome Database (HMDB) superclass level (E) or class level (F). The different colors represent different superclasses or class levels. (G) Only the top 10 enriched pathways are shown. The bars represent the enrichment ratios of genes.

To further characterize dynamic serum metabolite changes associated with TAO progression, OPLS‐DA was performed using untargeted metabolomics data from TAO patients with DON (n = 57) and without DON (n = 25). The resulting score plots showed clear separation between the two groups in both ESI+ and ESI– modes (Figure 2A), indicating distinct metabolic profiles at different stages of TAO. In ESI–mode, OPLS‐DA combined with Mann–Whitney U testing identified 19 differential metabolites with VIP ≥ 1 and *P* < 0.05; of these, 16 were upregulated and 3 were downregulated in non‐DON patients (Figure 2B). In ESI+ mode, 46 differential metabolites were detected, with 23 more abundant and 23 less abundant in non‐DON patients compared with those with DON (Figure 2B and Table S2). Heatmaps illustrating the relative abundance of these metabolites across disease states are shown in Figure 2C, and the top 10 differential metabolites are highlighted in Figure 2D. Hypoxanthine, previously implicated in B‐ and T‐cell proliferation in TAO, was among the top‐ranked metabolites.

Pie charts summarizing the classification of known differential metabolites according to HMDB annotations at the class and superclass levels are shown in Figure 2E,F. At the superclass level, the predominant groups included organic acids (23.53%), fatty acyls (17.65%), organoheterocyclic compounds (17.65%), sterol lipids (17.65%), and benzenoids (17.65%). At the class level, fatty acids and conjugates (17.65%) and amino acids and peptides (11.76%) were the most represented categories, with the remaining classes containing equal numbers of metabolites.

KEGG pathway enrichment analysis of all differential metabolites identified taurine and hypotaurine metabolism as the most significantly enriched pathway (Figure 2G and Table S6). Consistent with this finding, taurine is well recognized as an essential molecule for ocular health [24].

### 2.4 Quantitative Targeted Metabolomics Analyses of TAO and Healthy Control Samples

**FIGURE 3:**
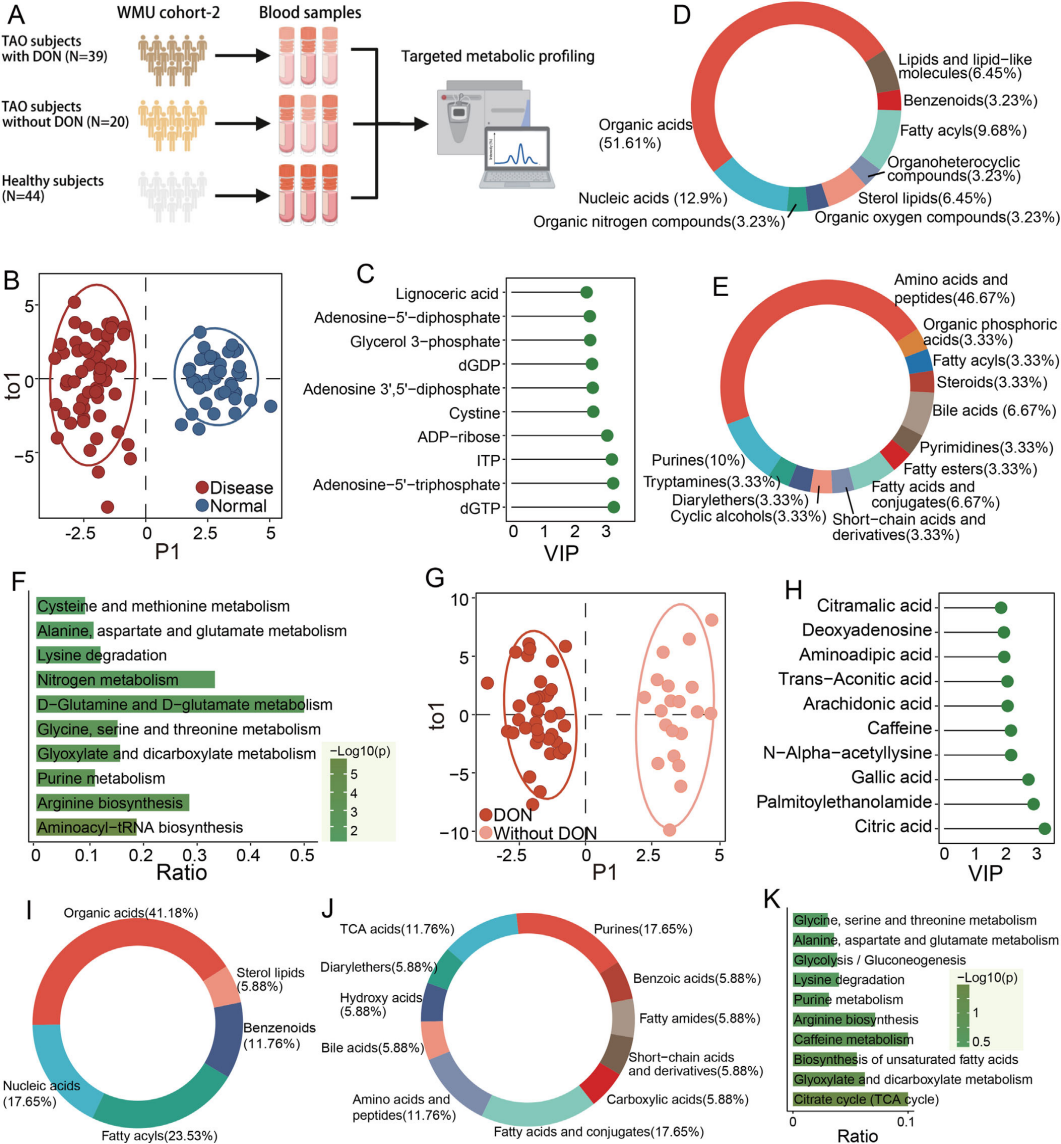
Characterization of dysregulated serum metabolomics in TAO using targeted metabolomics. (A) Workflow depicting the details of targeted metabolic profiling of the WMU cohort‐2. (B and G) Score plot of the orthogonal partial least squares–discriminant analysis (OPLS‐DA) model of disease samples (*n* = 59) and healthy samples (*n* = 44) (B) and without DON samples (*n* = 20) and with DON samples (*n* = 39) (G) of plasma samples based on targeted metabolomics. (C and H) The top 10 dysregulated known metabolites are shown for the disease versus normal (C) and with DON versus without DON (H). (D, E, I, and J) The proportions of chemical superclasses (D and I) and classes (E and J) of annotated metabolites in our study. (F and K) Enriched Kyoto Encyclopedia of Genes and Genomes (KEGG) pathways. The horizontal axis represents the gene ratio, calculated by dividing the number of genes hit in the pathway by the total number of genes in the term.

Based on the untargeted metabolomics analyses that characterized serum metabolic abnormalities associated with TAO onset and progression, targeted metabolomic profiling was performed to obtain deeper mechanistic insight (Figure 3A). OPLS‐DA was conducted on 103 samples (Table 2), and the resulting score plot demonstrated clear segregation between TAO patients and healthy controls (Figure 3B). Among the 40 metabolites differentially abundant at disease initiation, 16 (40%) were elevated in the TAO group and 24 (60%) were higher in controls (Figure S3A and Table S3). Corresponding heatmaps illustrate the relative expression patterns of these metabolites (Figure S3B). The top 10 metabolites were involved mainly in DNA or RNA synthesis, serving as substrates and/or energy sources (Figure 3C). At the superclass level, more than half were classified as organic acids, while amino acids and peptides represented the most frequent class, accounting for nearly half of these metabolites (Figure 3D,E). Pathway enrichment analysis confirmed that the breadth of detected pathways was sufficient to explain the observed metabolic shifts (Figure 3F and Table S7), with aminoacyl‐tRNA biosynthesis and arginine biosynthesis emerging as the most significantly enriched pathways.

Key metabolites associated with disease progression were further assessed using two‐dimensional OPLS‐DA score plots (Figure 3G), which showed distinct metabolic signatures in patients with and without DON. Comparative analysis identified eight downregulated and 14 upregulated metabolites in DON patients (Figure S3C and Table S4), with the upregulated metabolites being more abundant overall in the DON group (Figure S3D). VIP ranking highlighted citric acid as the most influential contributor (Figure 3H). According to HMDB superclass classifications, these differential metabolites were predominantly organic acids (41.18%) and fatty acyls (23.53%) (Figure 3I). At the class level, purines and fatty acids/conjugates were each the most represented categories (17.65%), followed by tricarboxylic acid (TCA) cycle intermediates and amino acids/peptides (each 11.76%) (Figure 3J). Consistent with these annotations, TCA cycle metabolism was the most significantly enriched pathway, suggesting a potential role in shaping the orbital microenvironment in TAO (Figure 3K and Table S8).

### 2.5 Integrated Metabolic Pathway Analysis Reveals Systemic Metabolic Dysregulation in TAO

**FIGURE 4:**
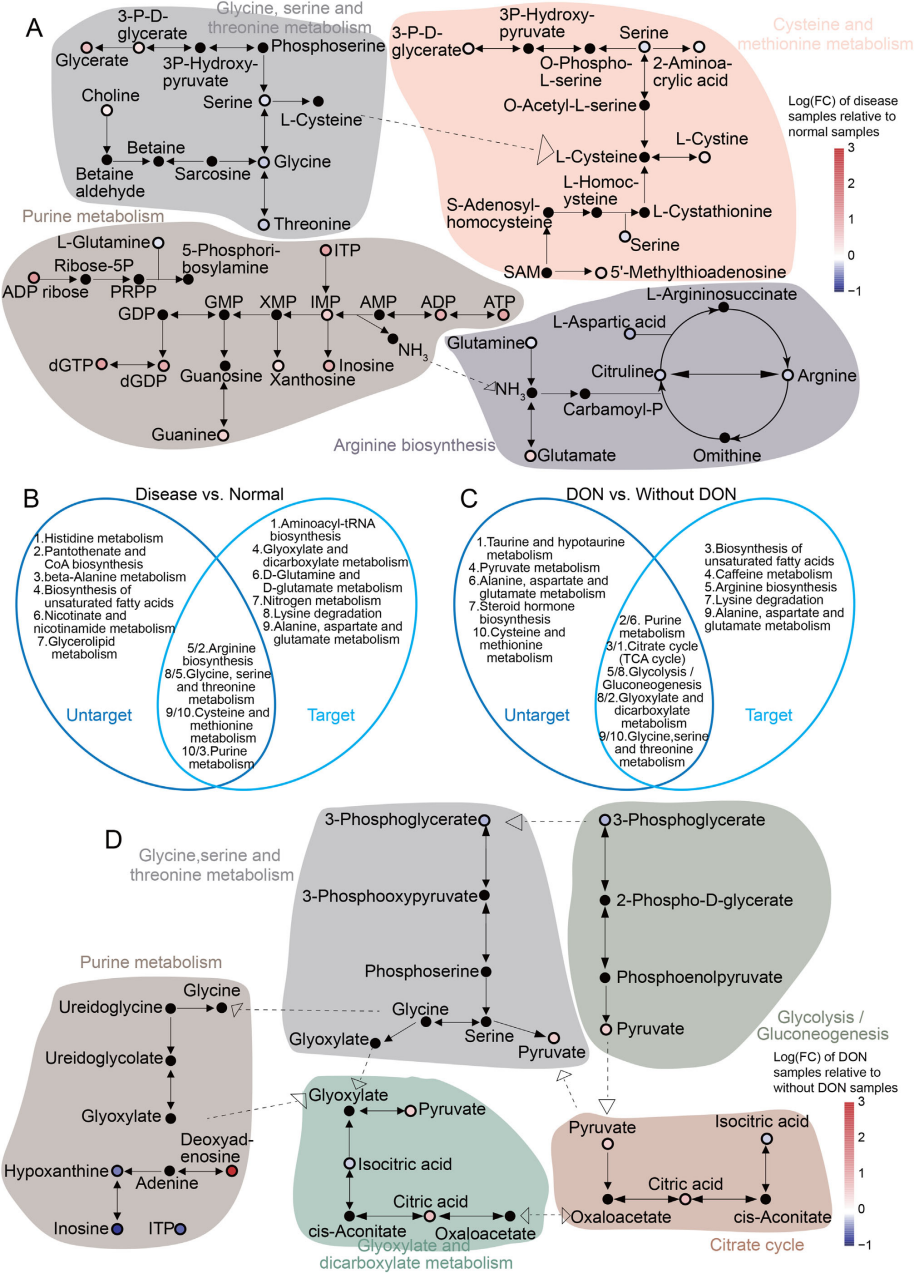
Identification of a metabolic pathway‐based paradigm to monitor dynamic changes in TAO. (A and B) Overlapping pathways of disease and normal (B) and with DON and without DON (C) between different cohorts. The numbers represent the pathways’ rank. Among the overlapping pathways, the first is the result of untargeted metabolism, and the second is the result of targeted metabolism. Diagram summarizing metabolites involved in shared pathways of disease and normal (A) and DON and non‐DON (D). Alteration of each metabolite is depicted as log ratios (fold‐change). Red, upregulated metabolites; blue, downregulated metabolites. FC, fold change.

Although the untargeted and targeted metabolomics analyses identified distinct sets of significantly altered metabolites, several of these metabolites converged on the same dysregulated metabolic pathways (Figure 4A). To better interpret the relevance of these pathways to TAO‐associated vasculopathy, both metabolomics datasets were integrated, and the top 10 dysregulated pathways identified in each analysis were compared.

As shown in Figure 4B, comparison of TAO patients with healthy controls revealed four pathways consistently altered in both datasets: arginine biosynthesis, cysteine and methionine metabolism, purine metabolism, and glycine, serine, and threonine metabolism. When comparing non‐DON and DON patients, five pathways were shared across the untargeted and targeted analyses, namely, the TCA cycle, glycolysis/gluconeogenesis, glyoxylate and dicarboxylate metabolism, purine metabolism, and glycine, serine, and threonine metabolism (Figure 4C). The significant differential metabolites identified in both datasets mapped directly to these shared dysregulated pathways (Figure 4D).

### 2.6 Oral L‐Arginine Supplementation Improves RCD and qCSF in DON Patients via the NO Pathway

**FIGURE 5:**
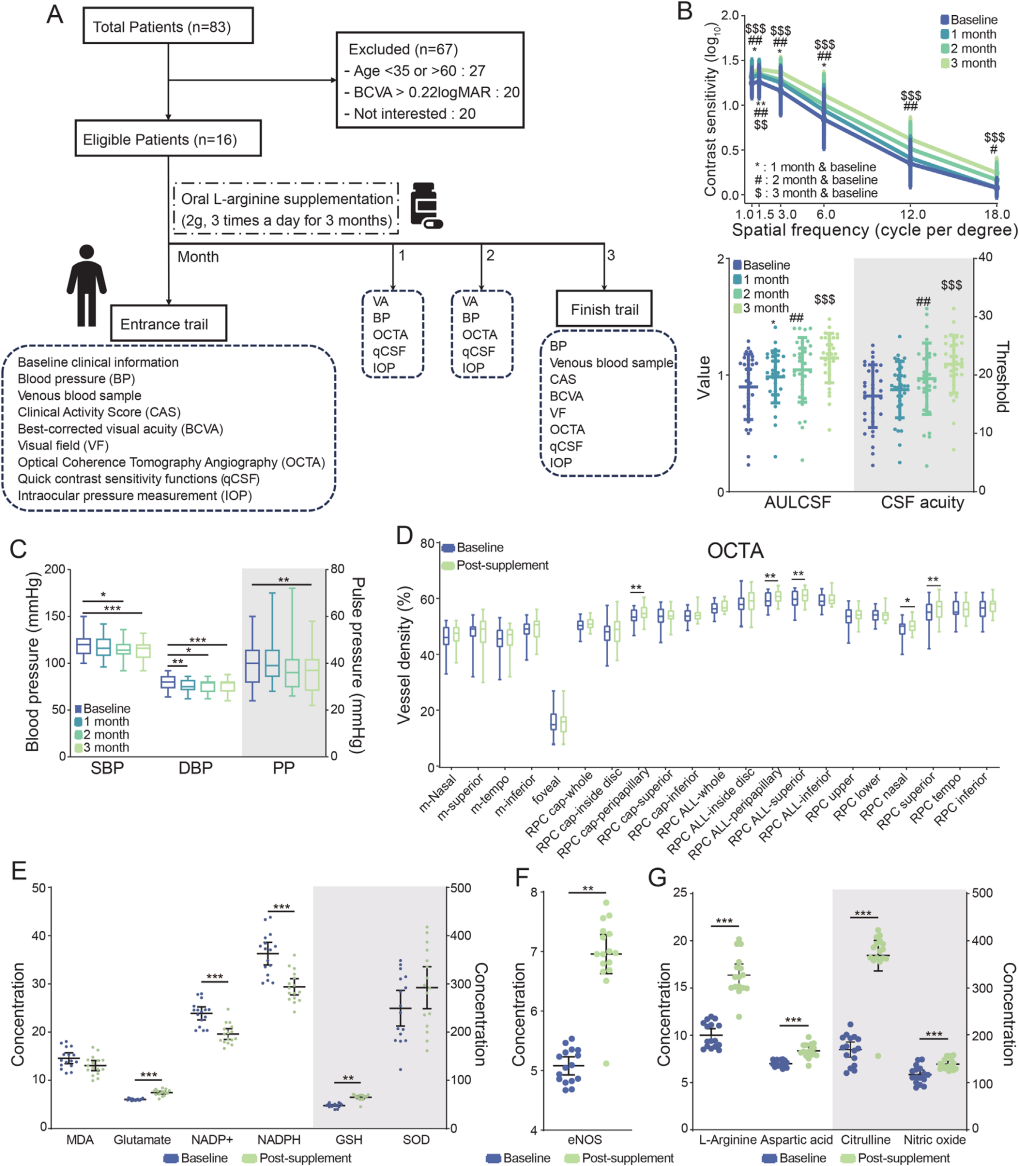
Oral L‐arginine supplementation enhances RCD and qCSF in early‐stage DON through the NO pathway. (A) Flowchart of the clinical trial (*n* = 16). (B) The changes in qCSF before and after supplementation are depicted, showing the mean ± standard deviation (SD) of the experimental results. (C–G) Illustrate the changes in BP, RCD, and serum molecular markers before and after supplementation, respectively. (C)–(G) presents the mean ± 95% confidence interval (CI). Symbol “*” denotes comparison with baseline at the first follow‐up. Symbol “#” denotes comparison with baseline at the second follow‐up (2 months). Symbol “$” denotes comparison with baseline at the third follow‐up (3 months). The number of symbols indicates the level of statistical significance: *, #, or $ for *p* < 0.05; **, ##, or $$ for *p* < 0.01; and ***, ###, or $$$ for *p* < 0.001. BCVA, best‐corrected visual acuity; BP, blood pressure; CAS, clinical activity score; DBP, diastolic blood pressure; DON, dysthyroid optic neuropathy; eNOS, endothelial nitric oxide synthase; GSH, glutathione; IOP, intraocular pressure; m, macular; MDA, malondialdehyde; NADP+, nicotinamide adenine dinucleotide phosphate; NADPH, nicotinamide adenine dinucleotide phosphate hydrogen; NO, nitric oxide; OCTA, optical coherence tomography angiography; PP, pulse pressure; qCSF, quick contrast sensitivity function; RPC, radial peripapillary capillary; SBP, systolic blood pressure; SOD, superoxide dismutase; VF, visual field.

The findings above indicate that dysregulated L‐arginine metabolism is closely linked to TAO pathogenesis. Oxidative stress–induced uncoupling of endothelial nitric oxide synthase (eNOS) reduces nitric oxide (NO) production, thereby contributing to disease development. Given that L‐arginine serves as a precursor for NO synthesis, it was hypothesized that oxidative stress may deplete L‐arginine and impair this pathway (see Graphical Abstract). To explore the clinical relevance of this mechanism, oral L‐arginine supplementation was administered for 3 months to 16 patients with early‐stage DON. Participants underwent monthly OCTA imaging, qCSF assessment, and blood pressure monitoring (Figure 5A).

Following supplementation, patients showed improvements in full‐spectrum qCSF performance (Figure 5B) and significant increases in RCD (Figure 5D), accompanied by reductions in systolic blood pressure (SBP), diastolic blood pressure (DBP), and PP (Figure 5C). Consistent with the mechanistic hypothesis, L‐arginine supplementation significantly elevated glutamic acid and glutathione levels while reducing NADP^+^ concentrations (Figure 5E). Similarly, both eNOS activity and plasma L‐arginine levels increased (Figure 5F,G).

**FIGURE 6:**
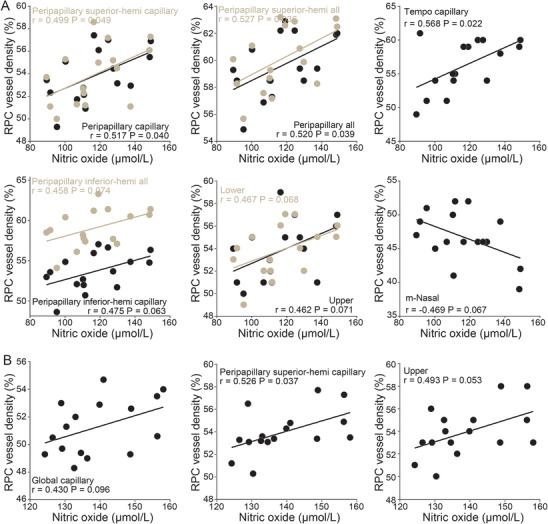
The correlation between vessel density and NO levels. (A) The correlation between vessel density and NO levels at baseline (*n* = 16). (B) The correlation between vessel density and NO levels after supplementation (*n* = 16). NO, nitric oxide; RPC, radial peripapillary capillary.

These results demonstrate that supplementation enhances NO production through L‐arginine–dependent antioxidant processes, including glutathione‐mediated restoration of eNOS coupling (Figure 5G). NO levels also correlated positively with RCD parameters (Figure 6).

These results suggest that L‐arginine supplementation improves RCD and visual function in DON by restoring the L‐arginine–NO pathway.

## 3 Discussion

In this study, the L‐arginine–NO pathway was identified as a central mediator of the RCD reduction observed in DON patients, primarily through mechanisms linked to oxidative stress. The initial clinical analyses demonstrated that decreased RCD is closely associated with systemic vascular abnormalities. Subsequent untargeted and targeted metabolomics further confirmed dysregulation of L‐arginine, the precursor of NO, in TAO patients. A clinical trial involving early‐stage DON patients showed that oral L‐arginine supplementation led to meaningful improvements in visual function, increased NO production, and reduced PP (see Graphical Abstract). These findings underscore the crucial roles of L‐arginine metabolism and NO signaling in DON pathophysiology and highlight promising avenues to improve vascular function and visual outcomes in affected patients.

Local vascular resistance, which governs blood flow, is modulated by vasoactive mediators released by endothelial cells and by regulatory inputs from glial and neural cells. Both our previous work and findings from other groups have shown that reduced RCD in DON correlates strongly with impaired visual function [14, 15, 25, 26]. This reduction has traditionally been attributed to mechanical compression resulting from increased orbital tissue volume, particularly at the orbital apex. However, several studies have reported that RCD continues to decline even after orbital decompression surgery [16, 17]. Consistent with these observations, it was found that reduced RCD, strongly associated with decreased visual quality as measured by qCSF [8], represents a DON‐related risk factor that is independent of orbital apex crowding.

Our analyses also revealed a consistent association between declining RCD and elevated PP [18], accompanied by higher rates of diabetes, ICAC, and significant dyslipidemia in DON patients (Table 1). These findings align with previous research linking lipid abnormalities to TAO onset [27, 28], and further reinforce the relationship between RCD decline and systemic vascular dysfunction. Taken together with recent single‐cell sequencing studies implicating endothelial cells in the pathogenesis of TAO [19], our results support the hypothesis that a shared systemic mechanism underlies both the reduction in RCD and the broader vascular abnormalities characteristic of DON.

Although our metabolomic analyses did not identify a single definitive biomarker, they did reveal substantial alterations in several metabolic pathways in TAO patients, including arginine, cysteine and methionine, purine, glycine, serine, and threonine metabolism. Compared with TAO patients without DON, those with DON showed additional disruptions in the TCA cycle, glycolysis/gluconeogenesis, glyoxylate and dicarboxylate metabolism, purine metabolism, and glycine, serine, and threonine metabolism.

As a direct precursor of NO, arginine plays a critical regulatory role in eNOS activity [29]. While eNOS is the predominant source of NO production, neuronal NOS (nNOS) and inducible NOS (iNOS) also contribute to NO generation and have been reported to influence retinal vasculature in adults [30, 31]. Under physiological conditions, specifically in the presence of oxygen, nicotinamide adenine dinucleotide phosphate hydrogen (NADPH), and tetrahydrobiopterin (BH_4_), eNOS catalyzes the conversion of L‐arginine to L‐citrulline, producing NO as a by‐product. Reduced NO bioavailability within vascular endothelial cells is believed to play a central role in the development of arteriosclerosis, vascular calcification, and adverse cardiovascular events [32, 33]. Clinical risk factors such as aging, hyperglycemia, hyperlipidemia, and smoking can impair endothelial function and diminish NO bioavailability, offering a plausible explanation for their association with DON. Given that RCD was correlated with NO levels both before and after L‐arginine supplementation (Figure 6), these findings suggest that diminished endothelial NO production plays a key role in the progression of RCD loss.

Oxidative stress is a central driver of TAO pathogenesis and an early therapeutic target, with particularly elevated levels observed in patients with DON [28]. Oxidative stress–induced eNOS uncoupling is strongly associated with endothelial injury, halting NO production and generating superoxide instead [34, 35]. Superoxide rapidly reacts with NO to form peroxynitrite, further depleting NO bioavailability. Within the ornithine cycle, L‐arginine is converted to urea, a process that promotes glutathione synthesis, an essential component of antioxidant defense [29]. Consistent with this mechanism, our retrospective cohort analysis revealed elevated urea levels in DON patients (Table 1).

NADPH, a critical antioxidant cofactor, is primarily produced through the citrate–pyruvate and pentose phosphate pathways. Under mitochondrial oxidative stress, the conversion of NADPH to NADP^+^ leads to BH_4_ depletion, resulting in eNOS uncoupling. As the NADP^+^/NADPH ratio increases, the pentose phosphate pathway becomes more active, generating NADPH and ribose‐5‐phosphate, both required for DNA repair, antioxidant responses, and purine metabolism. Declining NO levels subsequently activate iNOS, and the resulting NO inhibits aconitase‐dependent conversion of citrate to isocitrate within the citric acid–pyruvate cycle, thereby promoting NADPH generation.

Our metabolomic analyses further support these interactions, revealing that DON patients demonstrate abnormalities in purine metabolism, TCA cycle activity, and glycolysis/gluconeogenesis. These metabolic disruptions also help explain why hyperglycemia and hyperlipidemia emerge as DON‐associated risk factors. These results suggest that oxidative stress–driven L‐arginine depletion and eNOS uncoupling lead to substantial reductions in NO levels, ultimately contributing to RCD decline.

Various studies have demonstrated that L‐arginine enhances NO production by regulating eNOS activity, thereby promoting vasodilation. Oral L‐arginine supplementation has been shown to reduce blood pressure, particularly PP and SBP [36, 37, 38, 39], and NO itself possesses antioxidant properties. In our previous work, elevated PP was reported in TAO patients, and metabolomic analyses further revealed reduced levels of L‐arginine and citrulline. Building on these findings, the present clinical trial showed that oral L‐arginine supplementation in early‐stage DON patients lowered PP, increased RCD, and improved qCSF performance, providing clinical support for the mechanistic model proposed above.

The beneficial effects of supplementation can be interpreted through the lens of the “L‐arginine paradox,” the observation that exogenous L‐arginine can stimulate NO production despite intracellular concentrations already exceeding the Km of eNOS [40, 41]. This phenomenon may reflect compartmentalized intracellular L‐arginine pools or limited recycling of L‐arginine from citrulline [42]. Post‐supplementation analyses in our study revealed increased L‐arginine levels, enhanced eNOS activity, and elevated NO production. The strong correlations observed between NO and RCD suggest a direct link between the two, supporting a model in which oxidative stress–mediated L‐arginine depletion and eNOS uncoupling reduce NO availability, thereby contributing to RCD decline and the development of DON.

This study has several limitations. First, the L‐arginine supplementation component was an exploratory, proof‐of‐concept trial conducted without a placebo control group. As a result, although the observed improvements are promising, they should be interpreted cautiously and validated in future randomized, placebo‐controlled studies. Second, while our findings highlight the involvement of the L‐arginine–NO pathway in DON‐related vascular dysfunction, the precise mechanisms, including eNOS activity and post‐translational regulation, require further investigation. Third, the long‐term feasibility, safety, and efficacy of L‐arginine supplementation were not assessed in this study, and these factors will be essential to evaluate before considering clinical translation.

In conclusion, the reduced RCD and increased incidence of ICAC observed in DON patients point to a shared pathogenic mechanism driven by diminished endothelial NO bioavailability. These results suggest that oxidative stress–mediated L‐arginine depletion and eNOS uncoupling are key contributors to this decline. While oral L‐arginine supplementation appears promising as a therapeutic strategy for DON, its efficacy must be validated in larger, well‐controlled clinical trials.

## 4 Materials and Methods

### 4.1 Study Design and Patient Enrollment

This study included three distinct patient cohorts, all recruited from the Affiliated Eye Hospital of Wenzhou Medical University.

### 4.2 Cohort 1: Retrospective Cohort Study

To investigate vascular changes associated with DON, a cross‐sectional, retrospective analysis of all TAO patients hospitalized between January 2016 and December 2021 with complete medical records was conducted. Baseline characteristics are shown in Table 2. Electronic medical records were reviewed to collect demographic information (age, sex, weight, height, blood pressure, and medical history), systemic evaluations (blood pressure, glucose, lipid profile, thyroid function), ophthalmic assessments (best‐corrected visual acuity [BCVA], visual field [VF] testing, and optical coherence tomography angiography [OCTA]), imaging findings from orbital high‐resolution computed tomography (HRCT), and surgical data.

### 4.3 Cohort 2: Metabolomics Study

To identify circulating metabolites associated with TAO, untargeted (WMU cohort‐1) and targeted (WMU cohort‐2) metabolomic analyses were performed on blood samples from 141 TAO patients and 79 healthy controls. Key participant characteristics are listed in Table 2. The study design and data analysis workflow are illustrated in Figures 1A and 3A. Clinical data, including demographic information, imaging findings, and surgical history, were recorded using a dedicated Case Report Form (CRF). All ophthalmic evaluations were conducted by a single trained examiner (J.Y.) and included BCVA, exophthalmometry, clinical activity score (CAS), VF testing, and OCTA. Fasting venous blood samples were collected from all participants for metabolic profiling (see Section 4.11).

### 4.4 Cohort 3: Prospective L‐Arginine Supplementation Study

Patients with early‐stage DON presenting to the outpatient clinic were enrolled in a 3‐month prospective study evaluating oral L‐arginine supplementation (6 g/day, administered in three divided doses). The selected dose falls within the established safe adult range of 3–8 g/day [43] and exceeds the average daily endogenous turnover of approximately 5.4 g [44], ensuring adequate surplus to influence the targeted vascular pathway. Participants underwent monthly OCTA imaging, qCSF testing (as previously described [8]), and blood pressure monitoring. Pathway‐related biomarkers were assessed at baseline and after supplementation using patient blood samples (see Molecular Marker Detection). An overview of the study design is provided in Figure 5A. Since this was an exploratory trial, the final sample size was determined by the number of eligible patients who could be recruited. Although an initial target of 30 participants had been proposed during ethical review, only 16 patients were ultimately enrolled.

### 4.5 Inclusion and Exclusion Criteria

TAO was diagnosed by an orbit‐specialized ophthalmologist (Y.T.) according to the Bartley International Diagnostic Criteria [45]. In patients with eyelid retraction, TAO was diagnosed if any of the following were present: abnormal thyroid function, proptosis with globe protrusion ≥ 20 mm and an interocular difference > 2 mm, extraocular muscle involvement indicated by motility restriction or enlargement on orbital HRCT, or optic nerve dysfunction not attributable to other causes. In the absence of eyelid retraction, a TAO diagnosis required abnormal thyroid function in addition to at least one of the following: proptosis, extraocular muscle involvement, or optic nerve dysfunction unrelated to other ocular diseases.

TAO patient inclusion criteria were as follows: (1) age 20–65 years; (2) spherical equivalent refractive error between +1.0 D and −6.0 D; and (3) non‐active disease (CAS < 3/7 [46]). Exclusion criteria included (1) any history of ocular trauma or surgery; (2) systemic metabolic or cardiovascular disease, such as diabetes, hypertension, dyslipidemia, or heart failure; (3) use of systemic corticosteroids (oral or intravenous) within 3 months before enrollment; and (4) concurrent ocular conditions that could confound study outcomes, including but not limited to cataract, significant media opacity, glaucoma, retinopathies, or any other optic neuropathy known to impair visual function (e.g., qCSF) or alter retinal microvasculature (e.g., OCTA measurements).

### 4.6 Classification of TAO

DON was diagnosed when patients met the following criteria: (1) best‐corrected visual acuity (BCVA) worse than 0.1 logMAR; (2) visual field mean deviation (MD) less than −2 dB; (3) presence of optic disc abnormalities; and (4) a relative afferent pupillary defect in cases of unilateral involvement.

Early‐stage DON (Cohort 3) was defined by the following additional criteria: (1) confirmed diagnosis of TAO, (2) absence of significant orbital apex crowding on orbital HRCT, (3) abnormal qCSF results, and (4) BCVA ≤ 0.22 logMAR.

### 4.7 Clinical Activity Score

Disease activity was assessed using the Clinical Activity Score (CAS) [46], with scores ≥ 3 indicating active disease and lower scores reflecting inactive disease. The CAS is based on seven clinical signs and symptoms, each assigned one point: (1) spontaneous retrobulbar pain, (2) pain on eye movement, (3) conjunctival injection, (4) conjunctival edema, (5) lacrimal gland swelling, (6) eyelid edema, and (7) eyelid injection.

### 4.8 Assessment of the Retinal Microvasculature

An Optovue OCTA system was used to evaluate retinal microvascular status. The device operates at 70,000 A‐scans per second, with a central wavelength of 840 nm, and incorporates eye‐tracking technology to minimize motion artifacts, enabling precise quantification of retinal microvasculature. All scans were reviewed for quality by the same ophthalmologist, and any scans deemed suboptimal by the system were repeated.

Superficial retinal capillary plexus (SRCP) images were acquired with a 3.0 × 3.0 mm fovea‐centered scan. In comparison, radial peripapillary capillary (RPC) images were obtained using a 4.5 × 4.5 mm scan centered on the optic nerve head (ONH). Microvascular parameters were assessed using vessel density (VD), automatically calculated by the built‐in Optovue OCTA algorithm. All individuals involved in the OCTA workflow, including the examining technician, data analysts, and outcome assessors, were blinded to patient group assignments.

### 4.9 Evaluation of the Systemic Macrovasculature

Arterial calcification and PP are established indicators of arterial sclerosis [47, 48], and were used in this study to evaluate large‐vessel status in the patient cohort. The ICAC was assessed using HRCT as previously described [49]. In brief, the conventional HRCT bone window (window width: 1500 HU; window level: 250 HU) was used to evaluate calcification in the cervical siphon segment of the internal carotid artery, defined as the region extending from the petrous apex to the anterior clinoid process. Calcification was graded on a four‐level scale: (1) none, (2) mild (thin, discontinuous), (3) moderate (thin and continuous, or thick but discontinuous), and (4) severe (thick and continuous). PP was calculated as the difference between SBP and DBP.

### 4.10 Analysis of Molecular Markers

Vascular molecular profiling included assessments of blood glucose, lipid levels, oxidative stress and antioxidant markers, and components of the L‐arginine pathway. After overnight fasting, venous blood samples were collected and processed in the hospital laboratory to measure metabolic parameters, including fasting plasma glucose (FPG), glycated hemoglobin (HbA1c), triglycerides (TG), total cholesterol (TC), high‐density lipoprotein (HDL), and low‐density lipoprotein (LDL), as well as thyroid function markers (thyrotropin receptor antibodies [TRAb], thyroid‐stimulating hormone [TSH], free triiodothyronine [FT3], free thyroxine [FT4], total triiodothyronine [TT3], and total thyroxine [TT4]).

Plasma oxidative stress indicators, antioxidant‐related factors, and L‐arginine–related metabolites were quantified using standardized commercial assay kits (Wuhan Mosak Biotechnology, Wuhan, China). Superoxide dismutase (SOD) was measured via ELISA (Cat# 69–30016), while malondialdehyde (MDA; Cat# 69–98244) and reduced glutathione (GSH; Cat# 69–80232) were assessed using thiobarbituric acid–based colorimetry and enzymatic recycling methods, respectively. NADP^+^/NADPH ratios were determined using enzymatic cycling assays (Cat# 69–95130). Components of the L‐arginine pathway were quantified using competitive ELISAs for L‐arginine (Cat# 69–59247) and aspartic acid (Cat# 69–98699), and sandwich ELISAs for citrulline (Cat# 69–99737) and endothelial NO synthase (eNOS; Cat# 69–20140). NO levels were evaluated indirectly using the Griess reaction to detect stable nitrite/nitrate metabolites (Beijing Solarbio Science & Technology Co., Ltd., Cat# BC1475).

All venous blood samples were collected in EDTA‐containing tubes. After a 10‐ to 20‐min incubation, samples were centrifuged at 3000 rpm for 10 min, and the resulting plasma was analyzed in triplicate. Mean values were used for statistical analyses.

### 4.11 Metabolomic Analysis

#### 4.11.1 Metabolite Extraction

Blood samples were collected in the morning into EDTA‐K_2_ tubes and centrifuged within 4 h (10 min, 1500 rpm, 4°C). The resulting plasma was aliquoted into 2‐mL tubes and stored at −80°C. After all participants had been enrolled, metabolites were extracted by mixing 100 µL of serum with 500 µL of ice‐cold methanol containing L‐L‐chlorophenylalanine (0.04 µg/mL). The mixture was incubated at −20°C for 30 min, centrifuged (15 min, 16,500 rpm, 4°C), and 500 µL of the supernatant was vacuum dried at 16°C (Thermo Scientific). Dried extracts were reconstituted in 180 µL of water for LC‐MS analysis. QC samples were prepared by pooling 10 µL aliquots from each metabolite extract.

### 4.12 Untargeted Metabolomics Profiling

Chromatographic separation was carried out using an Ultimate 3000 UPLC system equipped with an Acquity HSS T3 column. For positive ion mode, the mobile phase consisted of 0.1% formic acid in water (A) and 0.1% formic acid in methanol (B). For negative ion mode, 6.5 mM NH_4_HCO_3_ (A) and methanol (B) were used. Samples were separated at a flow rate of 0.3 mL/min using a gradient elution from 2% to 98% B over 17 min.

Mass spectrometric analysis was performed on a Thermo Scientific Orbitrap Fusion Lumos operating in both positive and negative electrospray ionization modes. Full MS scans were acquired at a resolution of 120,000 across an *m*/*z* range of 70–1050. MS/MS spectra were collected with a 0.8‐s cycle time using multiple collision energies and resolutions. Ionization source parameters included spray voltages of +3.8 kV and −2.5 kV, and a capillary temperature of 320°C.

### 4.13 Targeted Metabolomics Profiling

Targeted metabolomics analyses were performed by Shanghai Applied Protein Technology Co., Ltd. using a UHPLC system (1290 Infinity LC, Agilent Technologies) coupled to a QTRAP mass spectrometer (6500+, Sciex). Analyte separation was achieved using both HILIC (Waters UPLC BEH Amide, 2.1 × 100 mm, 1.7 µm) and C18 columns (Waters UPLC BEH C18, 2.1 × 100 mm, 1.7 µm).

For HILIC separation, the column was maintained at 35°C with a 2 µL injection volume. The mobile phases consisted of water containing 2 mM ammonium formate and 10% acetonitrile (A) and 0.4% formic acid in methanol (B). The gradient program ranged from 85% to 50% B over 15.5 min, then returned to 85% B by 23 min, with a constant flow rate of 300 µL/min.

Reversed‐phase liquid chromatography (RPLC) was conducted at 40°C using mobile phases of 5 mM ammonium acetate with 0.2% NH_3_·H_2_O in water (A) and 99.5% acetonitrile with 0.5% NH_3_·H_2_O (B). The gradient progressed from 5% to 100% B over 11 min, then returned to 5% B by 16 min, at a flow rate of 400 µL/min. Samples were maintained at 4°C throughout analysis.

A 6500+ QTRAP system operated in positive/negative switching mode was used for multiple reaction monitoring (MRM)‐based quantitative analysis. System stability and reproducibility were verified using QC samples.

### 4.14 Bioinformatics Analyses

Stability was evaluated using PCA implemented via the ggfortify R package. Global metabolic differences were assessed using OPLS‐DA with the ropls package. Significantly differentially expressed metabolites (DEMs) were identified based on a Mann–Whitney *U* test (*p* < 0.05) and an OPLS‐DA VIP score ≥ 1. Metabolite classification and pathway enrichment analyses were conducted using MetaboAnalyst v5.0, with annotations derived from KEGG.

### 4.15 Statistics

Statistical analyses were performed using IBM SPSS 25.0. Normality of continuous variables was assessed using the Shapiro–Wilk test. Normally distributed data are presented as mean ± standard deviation, while non‐normally distributed data are expressed as median (interquartile range). Categorical variables are reported as percentages. Comparisons among the normal, non‐DON, and DON groups were conducted using ANOVA or Kruskal–Wallis tests with appropriate post hoc analyses. Paired *t*‐tests or Wilcoxon signed‐rank tests were applied to evaluate pre‐ and post‐supplementation differences within individuals. Chi‐square or Fisher's exact tests were used for categorical variable comparisons. Correlations between clinical variables were assessed using Pearson, Spearman, or Kendall's tau‐b coefficients. For analysis, BCVA values were converted to logMAR. A *p* value < 0.05 was considered statistically significant.

## Author Contributions

Conceptualization: Yunhai Tu, Meng Zhou, Kang Zhang, and Wencan Wu. Writing – original draft: Yunhai Tu, Congcong Yan, Lu Chen, and Weijie Liu. Writing – review and editing: Mengyuan Gao, Meng Zhou, Kang Zhang, and Wencan Wu. Funding acquisition: Yunhai Tu and Wencan Wu. Data curation: Congcong Yan, Lu Chen, Xiaozhou Hu, Wei Rao, Jiayi Zhang, Junye Zhu, and Hui Wu. Visualization: Congcong Yan and Meng Zhou. Formal analysis: Congcong Yan, Lu Chen, Weijie Liu, Mengyuan Gao, and Wei Rao. Resources: Lu Chen, Weijie Liu, Xiaozhou Hu, Mengyuan Gao, Wei Rao, Jiayi Zhang, Junye Zhu, and Hui Wu. All authors have read and approved the final manuscript.

## Funding

This study was supported by the National Natural Science Foundation of China (Grant No. 82471126), the Natural Science Foundation of Zhejiang Province (Grant No. LTGD24H120001), the Zhejiang Provincial Medical and Health Science and Technology Plan Project (Grant No. WKJ‐ZJ‐2336), the Scientific Research Found of Zhejiang Privincial Education Department (Grant No. Y202249996), and the National Key Research and Development Program of China (2021YFA1101200).

## Conflicts of Interest

Author Kang Zhang is an editorial board member of MedComm. Author Kang Zhang was not involved in the journal's review of or decisions related to this manuscript. The other authors declare no conflicts of interest.

## Ethics Statement

This study was approved by the ethics committee of the Affiliated Eye Hospital of Wenzhou Medical University (approval number: 2020‐106‐K‐93‐01, 2023‐047‐K‐39‐01, and 2024‐209‐K‐174‐01). This study was conducted in compliance with the Declaration of Helsinki and all applicable ethical guidelines.

## Consent

The requirement for informed consent was waived for the retrospective analysis of Cohort 1, while written consent was obtained from patients in Cohorts 2 and 3.

## Supporting information




**Figure S1**. Correlation between pulse pressure (PP) and macular superficial retinal capillary density (RCD) in TAO patients (n = 357). A‐E represent the capillary density in the superficial retinal layer of the macular region, specifically in the parafoveal, superior, inferior, nasal, and temporal regions.
**Figure S2**. Characterization of dysregulated serum metabolomics in TAO using untargeted metabolomics. (A) Relative standard deviation (RSD) distribution of metabolic features in quality control (QC) samples. (B) Venn diagrams demonstrating the overlapping between differential metabolites identified from univariate and multivariate analysis in Electrospray Ionization negative (ESI‐) mode and Electrospray Ionization positive (ESI+) mode. Red: upregulate in TAO group, blue: upregulate in control group. (C) Heatmap of differentially expressed metabolites (DEMs) between TAO patients and healthy controls.
**Figure S3**. Characterization of dysregulated serum metabolomics in TAO with/without DON using targeted metabolomics. (A) Venn diagram showing differentially abundant metabolites between TAO patients and healthy controls. (B) Heatmap of relative abundance of differentially expressed metabolites (DEMs) in disease versus control groups. (C) Comparison of DEMs between TAO patients with and without DON. (D) Heatmap of DEMs in DON versus non‐DON subgroups
**Table S1**. Results of DEMs in untargeted metabolomics (Normal Group vs. Disease Group)
**Table S2**. Results of DEMs in untargeted metabolomics (Non‐DON Group vs. DON Group)
**Table S3**: Results of DEMs in targeted metabolomics (Normal Group vs. Disease Group)
**Table S4**: Results of DEMs in targeted metabolomics (Non‐DON Group vs. DON Group)
**Table S5**: Pathway enrichment analysis of untargeted metabolomics (Normal Group vs. Disease Group)
**Table S6**: Pathway enrichment analysis of untargeted metabolomics (Non‐DON Group vs. DON Group)
**Table S7**: Pathway enrichment analysis of targeted metabolomics (Normal Group vs. Disease Group)
**Table S8**: Pathway enrichment analysis of targeted metabolomics (Non‐DON Group vs. DON Group)

## Data Availability

The data reported in this paper have been deposited in the OMIX, China National Center for Bioinformation/Beijing Institute of Genomics, Chinese Academy of Sciences (https://ngdc.cncb.ac.cn/omix: accession no. OMIX013067). The clinical data from the retrospective and prospective cohorts generated and/or analysed during the current study are not publicly available due to the inclusion of sensitive personal health information of patients, which could compromise individual privacy, but are available from the corresponding author on reasonable request.
