# Supplementary material for: Retinal Capillary Density Reduction Contributes to Dysthyroid Optic Neuropathy via an L‐Arginine‐NO Pathway: A Metabonomics and Clinical Trial Study
Source: MedComm (2020). 2026 Mar 4;7(3):e70652. doi: 10.1002/mco2.70652 (PMC12960065; doi:10.1002/mco2.70652)
Supplement: Supplementary file 1 — Figure S1. Correlation between pulse pressure (PP) and macular superficial retinal capillary density (RCD) in TAO patients (n = 357). A‐E represent the capillary density in the superficial retinal layer of the macular region, specifically in the parafoveal, superior, inferior, nasal, and temporal regions. Figure S2. Characterization of dysregulated serum metabolomics in TAO using untargeted metabolomics. (A) Relative standard deviation (RSD) distribution of metabolic features in quality control (QC) samples. (B) Venn diagrams demonstrating the overlapping between differential metabolites identified from univariate and multivariate analysis in Electrospray Ionization negative (ESI‐) mode and Electrospray Ionization positive (ESI+) mode. Red: upregulate in TAO group, blue: upregulate in control group. (C) Heatmap of differentially expressed metabolites (DEMs) between TAO patients and healthy controls. Figure S3. Characterization of dysregulated serum metabolomics in TAO with/without DON using targeted metabolomics. (A) Venn diagram showing differentially abundant metabolites between TAO patients and healthy controls. (B) Heatmap of relative abundance of differentially expressed metabolites (DEMs) in disease versus control groups. (C) Comparison of DEMs between TAO patients with and without DON. (D) Heatmap of DEMs in DON versus non‐DON subgroups Table S1. Results of DEMs in untargeted metabolomics (Normal Group vs. Disease Group) Table S2. Results of DEMs in untargeted metabolomics (Non‐DON Group vs. DON Group) Table S3: Results of DEMs in targeted metabolomics (Normal Group vs. Disease Group) Table S4: Results of DEMs in targeted metabolomics (Non‐DON Group vs. DON Group) Table S5: Pathway enrichment analysis of untargeted metabolomics (Normal Group vs. Disease Group) Table S6: Pathway enrichment analysis of untargeted metabolomics (Non‐DON Group vs. DON Group) Table S7: Pathway enrichment analysis of targeted metabolomics (Normal Group vs. Disease Group) Table S8: P [file MCO2-7-e70652-s001.docx]

**Supplementary Information for: Retinal capillary density reduction contributes to dysthyroid optic neuropathy via an L-arginine-NO Pathway: A metabonomics and clinical trial study**

**Running title: L-arginine-NO Pathway Targets RCD in DON**

**Yunhai Tu^1,2#^, Congcong Yan^1,2,3#^, Lu Chen^1,2#^, Weijie Liu^1,2^, Xiaozhou Hu^1,2^, Mengyuan Gao^1,2^, Wei Rao^1,2^, Jiayi Zhang^1,2^, Junye Zhu^1,2^, Hui Wu^1,2^, Kang Zhang^4*^, Meng Zhou^1,2,3*^, Wencan Wu^1,2,5,6*^**

^1^State Key Laboratory of Ophthalmology, Optometry and Visual Science, Eye Hospital, Wenzhou Medical University, Wenzhou, China

^2^National Clinical Research Center for Ocular Diseases, Eye Hospital, Wenzhou Medical University, Wenzhou, China.

^3^School of Biomedical Engineering, Eye Hospital, Wenzhou Medical University, Wenzhou, China

^4^Institute for Advanced Study on Eye Disease and Health, Wenzhou Medical University, Wenzhou, Zhejiang, China

^5^Zhejiang Key Laboratory of Core Technologies for Reconstruction of Ocular-Cerebral Neural Pathway Functions, Zhejiang, China

^6^Oujiang Laboratory (Zhejiang Lab for Regenerative Medicine, Vision and Brain Health), Zhejiang, China

#These authors contributed equally.

*Correspondence should be addressed to: Wencan Wu, wuwencan@wmu.edu.cn; Meng Zhou, zhoumeng@wmu.edu.cn; Kang Zhang, kang.zhang@gmail.com


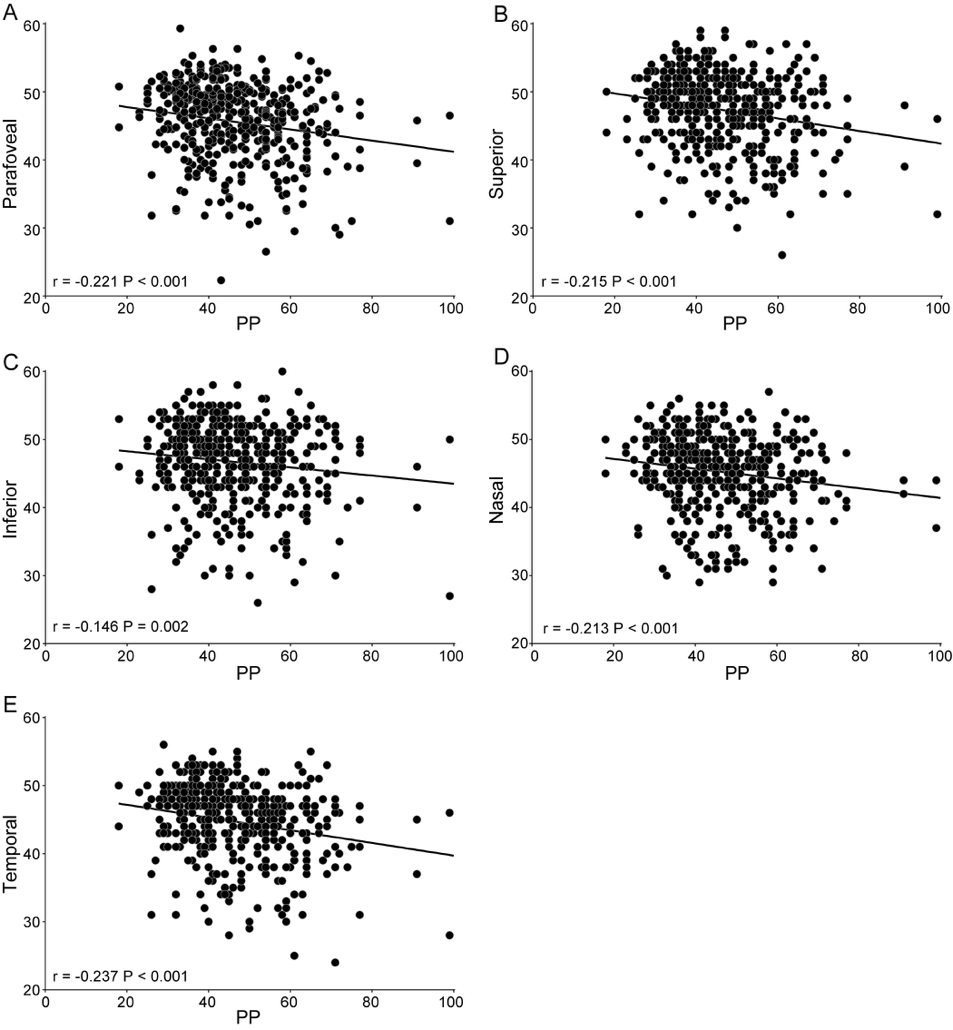


**Figure S1. Correlation between pulse pressure (PP) and macular superficial retinal capillary density (RCD) in TAO patients (n=357).** A-E represent the capillary density in the superficial retinal layer of the macular region, specifically in the parafoveal, superior, inferior, nasal, and temporal regions.


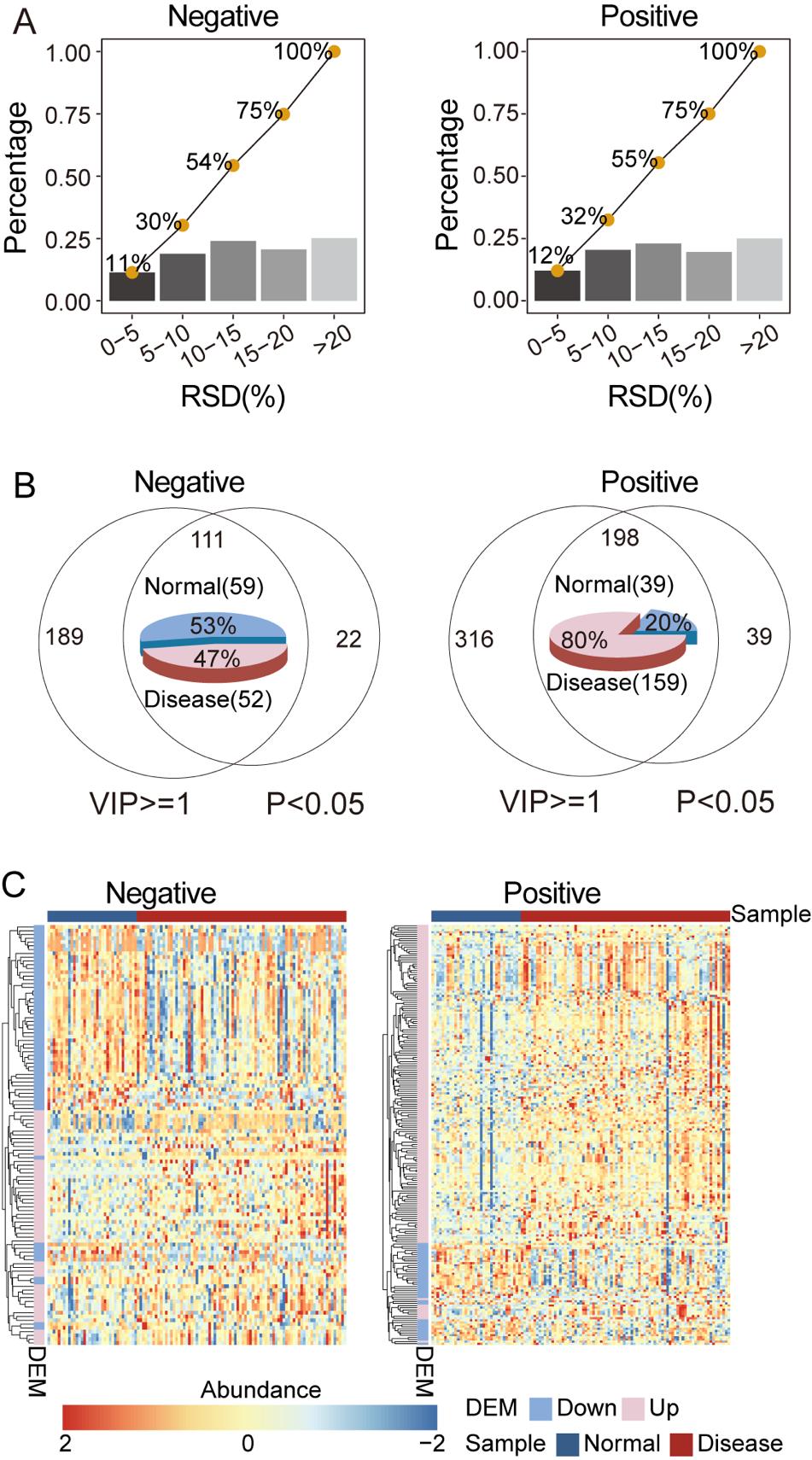


**Figure S2. Characterization of dysregulated serum metabolomics in TAO using untargeted metabolomics.** (A) Relative standard deviation (RSD) distribution of metabolic features in quality control (QC) samples. (B) Venn diagrams demonstrating the overlapping between differential metabolites identified from univariate and multivariate analysis in Electrospray Ionization negative (ESI-) mode and Electrospray Ionization positive (ESI+) mode. Red: upregulate in TAO group, blue: upregulate in control group. (C) Heatmap of differentially expressed metabolites (DEMs) between TAO patients and healthy controls.


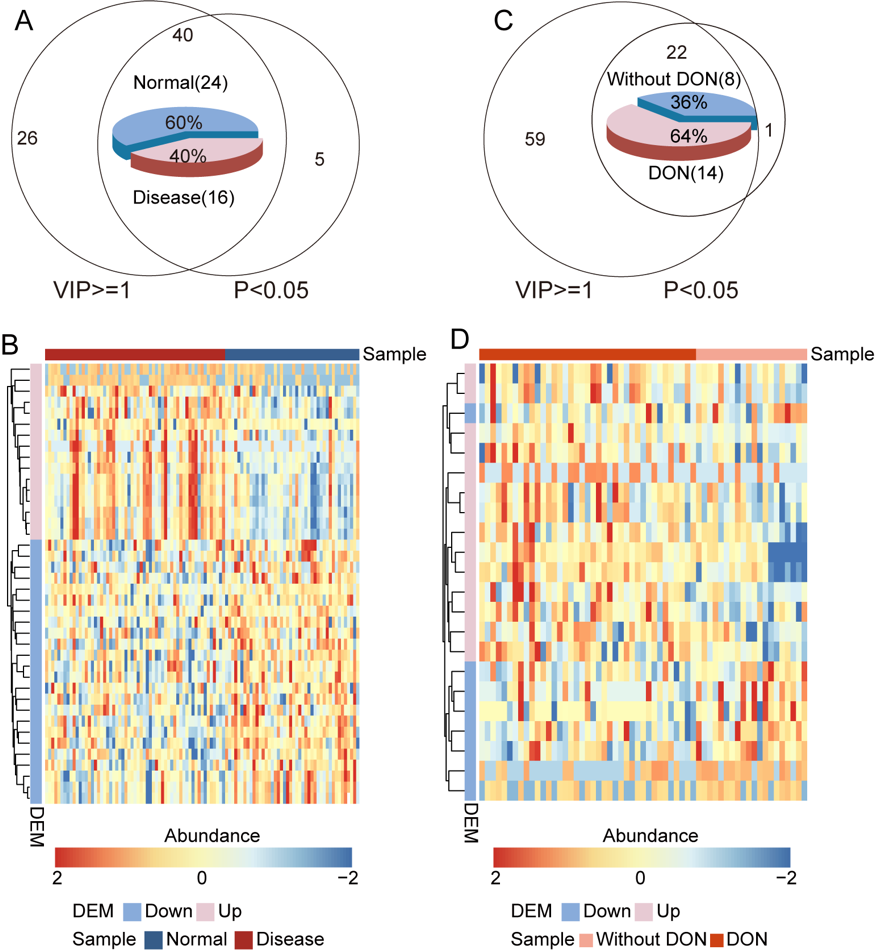


**Figure S3. Characterization of dysregulated serum metabolomics in TAO with/without DON using targeted metabolomics.** (A) Venn diagram showing differentially abundant metabolites between TAO patients and healthy controls. (B) Heatmap of relative abundance of differentially expressed metabolites (DEMs) in disease versus control groups. (C) Comparison of DEMs between TAO patients with and without DON. (D) Heatmap of DEMs in DON versus non-DON subgroups.

| **Table S1. Results of DEMs in untargeted metabolomics (Normal Group vs. Disease Group)** | | | | | |
| --- | --- | --- | --- | --- | --- |
| DEM | *P* | FDR | Pattern | logFC | sig |
| (-)-ALPHA-CEDRENE | 0.000654699 | 0.068661586 | negative | -0.755327868 | normal |
| (-)-MENTHYLACETATE | 0.02957881 | 0.251927477 | negative | -0.413508525 | normal |
| (11E)-2-HEPTYL-10-OXO-11-NONADECENOIC ACID | 0.036407361 | 0.270316604 | negative | -0.534276591 | normal |
| (1S,2R,5S)-2-ISOPROPYL-5-METHYLCYCLOHEXYL 3-OXOBUTANOATE | 0.003062122 | 0.107046668 | negative | -0.637360312 | normal |
| (2R)-2,3-DIHYDROXYPROPANOIC ACID | 1.36152E-06 | 0.001142313 | negative | 0.705232358 | disease |
| (2S)-1-HYDROXY-3-(PENTADECANOYLOXY)-2-PROPANYL (9Z,12Z,15Z)-9,12,15-OCTADECATRIENOATE | 0.004477817 | 0.129547875 | negative | -0.819202826 | normal |
| (5Z,9Z)-2-METHOXY-5,9-HEXACOSADIENOIC ACID | 0.008437618 | 0.15379105 | negative | -0.550025381 | normal |
| [FA(18:4)]6Z_9Z_12Z_15Z-OCTADECATETRAENOICACID | 0.020744884 | 0.21487602 | negative | -0.594821557 | normal |
| [FAMETHYL(18:0)]9_10-METHYLENE-9-OCTADECENOICACID | 0.04581363 | 0.305060598 | negative | -0.506678338 | normal |
| [FAOXO_AMINO(6:0)]3-OXO-5S-AMINO-HEXANOICACID | 0.035878562 | 0.270316604 | negative | 0.52117632 | disease |
| [SIMILAR TO: 5-OXOETE; ΔMASS: 257.1036 DA] | 0.018264267 | 0.207077297 | negative | 0.251340208 | disease |
| [SIMILAR TO: ARACHIDONIC ACID; ΔMASS: 197.0459 DA] | 0.001027547 | 0.070868501 | negative | 1.583119878 | disease |
| [SIMILAR TO: ARACHIDONIC ACID; ΔMASS: 239.0566 DA] | 0.012947075 | 0.178075339 | negative | 0.282973159 | disease |
| [SIMILAR TO: CYSTEINYLGLYCINE; ΔMASS: 133.0201 DA] | 0.041472513 | 0.285208513 | negative | 0.310528251 | disease |
| [SIMILAR TO: D-(+)-TRYPTOPHAN; ΔMASS: 162.0534 DA] | 0.037484906 | 0.273476834 | negative | -0.664277829 | normal |
| [SIMILAR TO: ETHYL PARABEN; ΔMASS: 80.0117 DA] | 0.007457206 | 0.144740875 | negative | 0.573336203 | disease |
| [SIMILAR TO: L-HISTIDINE; ΔMASS: 112.9977 DA] | 0.025796689 | 0.237839804 | negative | 0.184320961 | disease |
| [ST(2:0)]ANDROSTAN-3BETA_17BETA-DIOL | 0.000964331 | 0.070868501 | negative | -0.740842631 | normal |
| 1-ARACHIDONOYL-SN-GLYCERO-3-PHOSPHOCHOLINE | 0.040881743 | 0.285208513 | negative | 0.204391206 | disease |
| 1-ARACHIDONOYL-SN-GLYCERO-3-PHOSPHOETHANOLAMINE | 0.001795337 | 0.083682647 | negative | 2.137321793 | disease |
| 1-LINOLEOYL-SN-GLYCERO-3-PHOSPHOETHANOLAMINE | 0.023151385 | 0.223264506 | negative | 1.768722788 | disease |
| 1-PALMITOYLGLYCEROL3-PHOSPHATE | 0.009048217 | 0.15379105 | negative | -2.304913821 | normal |
| 1-PALMITOYLGLYCEROPHOSPHOCHOLINE | 0.019470086 | 0.214149982 | negative | -1.789608364 | normal |
| 1-STEAROYL-2-HYDROXY-SN-GLYCERO-3-PE | 0.005290278 | 0.135424411 | negative | -1.216528697 | normal |
| 10-UNDECENOIC ACID | 0.017686256 | 0.203270808 | negative | -0.431273573 | normal |
| 12-HYDROXYDODECANOICACID | 0.005692362 | 0.136454061 | negative | -0.595037406 | normal |
| 19(R)-HYDROXY PROSTAGLANDIN A2 | 0.007590701 | 0.144740875 | negative | 0.820204301 | disease |
| 2-AMINOACRYLATE | 0.01273216 | 0.178038043 | negative | -0.125124083 | normal |
| 2-ARACHIDONOYLGLYCEROL | 0.002194036 | 0.087656981 | negative | -0.542803651 | normal |
| 2-HYDROXY-3-(PHOSPHONOOXY)PROPYL (4Z,7Z,10Z,13Z,16Z)-4,7,10,13,16-DOCOSAPENTAENOATE | 0.038589472 | 0.279108334 | negative | 0.329306243 | disease |
| 2-HYDROXYCEROTIC ACID | 0.002328593 | 0.088804071 | negative | -1.636222248 | normal |
| 2-HYDROXYTRICOSANOIC ACID | 0.045170957 | 0.303187464 | negative | -0.451840787 | normal |
| 2-METHOXY-17BETA-ESTRADIOL 3-SULFATE | 0.012106242 | 0.172154863 | negative | 0.507341524 | disease |
| 2-METHYL-6-OXOHEPTA-2_4-DIENAL | 0.009206903 | 0.15379105 | negative | 0.296489489 | disease |
| 2,2'-(2,2'-BITHIENE-5,5'-DIYL)BIS(PENTAMETHYLDISILANE) | 0.010038184 | 0.158479305 | negative | -0.198000205 | normal |
| 2,2,7,7-TETRAMETHYL-4-[(OCTADECYLOXY)METHYL]-3,6-DIOXA-2,7-DISILAOCTANE | 0.030027113 | 0.251927477 | negative | -0.294258904 | normal |
| 2,3,4,5,6-PENTAHYDROXY-N-(2-HYDROXYETHYL)HEXANAMIDE | 2.28253E-05 | 0.006383468 | negative | 2.511717206 | disease |
| 22-TRICOSENOIC ACID | 0.01740331 | 0.202796901 | negative | -0.525040051 | normal |
| 2287460 | 0.009048217 | 0.15379105 | negative | 0.398733 | disease |
| 2512 | 0.02351424 | 0.224186897 | negative | -0.184040682 | normal |
| 2515 | 0.00300345 | 0.107046668 | negative | -0.697002661 | normal |
| 2518 | 0.009368068 | 0.15379105 | negative | -0.436486086 | normal |
| 3-(4,4-DIMETHYL-4-AMINOBUTYL)-INDOLE | 0.001267017 | 0.070868501 | negative | -0.527116398 | normal |
| 3-[6-({6-O-[(2R,3R,4R)-3,4-DIHYDROXY-4-(HYDROXYMETHYL)TETRAHYDRO-2-FURANYL]-BETA-D-GLUCOPYRANOSYL}OXY)-1-BENZOFURAN-5-YL]PROPANOIC ACID | 0.005487998 | 0.135424411 | negative | 0.711624124 | disease |
| 3-HYDROXYDECANOIC ACID | 0.016849302 | 0.201950919 | negative | -0.400536443 | normal |
| 3-METHYLHIPPURIC ACID | 0.031879199 | 0.261358415 | negative | 0.807488765 | disease |
| 3-OXOTETRADECANOIC ACID | 0.006699538 | 0.140522812 | negative | -0.660644316 | normal |
| 3,4-DIHYDROXYPHENYLGLYCOL O-SULFATE | 0.032841407 | 0.261358415 | negative | 0.30587725 | disease |
| 3222 | 0.001267017 | 0.070868501 | negative | -0.806516489 | normal |
| 4-ALLYL-2-HYDROXY-6-METHOXYPHENYL HYDROGEN SULFATE | 0.000654699 | 0.068661586 | negative | -1.515936734 | normal |
| 4-FORMYL-2-METHOXYPHENYL HYDROGEN SULFATE | 0.009531746 | 0.15379105 | negative | -1.554181682 | normal |
| 5-HYDROXYDIHYDRO-2,4(1H,3H)-PYRIMIDINEDIONE | 0.004154449 | 0.129095669 | negative | 0.708794578 | disease |
| 5ALPHA-ANDROSTANE-3ALPHA,17BETA-DIOL DISULFATE | 0.019782196 | 0.214149982 | negative | 0.728823491 | disease |
| 6-DEOXOTEASTERONE | 0.007457206 | 0.144740875 | negative | -0.928603513 | normal |
| 609975 | 0.001190254 | 0.070868501 | negative | 0.638266821 | disease |
| ABIETIC ACID | 0.049143655 | 0.312360047 | negative | -0.076743936 | normal |
| ANDROSTERONE SULFATE | 0.027842696 | 0.24851087 | negative | 0.53999359 | disease |
| APM | 0.02869945 | 0.251927477 | negative | 0.249236359 | disease |
| ARACHIDONIC ACID | 0.041472513 | 0.285208513 | negative | -0.442040074 | normal |
| CYCLOPENTA[CD]PYRENE | 0.02140914 | 0.216412877 | negative | 0.712630137 | disease |
| CYCLOPENTADECANOLIDE | 0.030027113 | 0.251927477 | negative | -0.493295125 | normal |
| CYPRODENATE | 0.014785093 | 0.191067005 | negative | 0.385985097 | disease |
| D-IDITOL | 0.001689416 | 0.083377666 | negative | 0.914506674 | disease |
| DICYCLOHEXYLCARBODIIMIDE | 0.020419545 | 0.214149982 | negative | -0.581453567 | normal |
| DIMETHYL THIOPHOSPHATE | 0.006580216 | 0.140522812 | negative | -0.196154994 | normal |
| DL-MANDELIC ACID | 0.030027113 | 0.251927477 | negative | -0.733627033 | normal |
| DOCOSAHEXAENOIC ACID | 0.000866918 | 0.070868501 | negative | -0.863144542 | normal |
| DOCOSAPENTAENOIC ACID | 0.016849302 | 0.201950919 | negative | -0.776413683 | normal |
| EBELACTONE B | 0.022440239 | 0.22149836 | negative | -0.634166207 | normal |
| EICOSAPENTAENOIC ACID | 0.011903748 | 0.172154863 | negative | -0.709852543 | normal |
| EPINEPHRINE 4-SULFATE | 0.016849302 | 0.201950919 | negative | 0.425272561 | disease |
| ETHOPABATE | 0.000210966 | 0.044250022 | negative | 0.702310097 | disease |
| FERULIC ACID 4-SULFATE | 0.00623363 | 0.140522812 | negative | 0.98033666 | disease |
| FURAN FATTY ACID F6 | 0.001376485 | 0.072179436 | negative | -0.822330207 | normal |
| GERANYLACETATE | 0.007864136 | 0.146622449 | negative | -0.535695234 | normal |
| GLU-THR | 0.008891978 | 0.15379105 | negative | -0.12320587 | normal |
| GLY-PRO(GLYCYLPROLINE) | 1.9459E-05 | 0.006383468 | negative | -1.264273078 | normal |
| GLYCYLPROLINE | 0.015030301 | 0.191067005 | negative | -0.346835828 | normal |
| HIS-ASP | 0.049143655 | 0.312360047 | negative | 0.249015809 | disease |
| HOMOCYSTEINE | 0.033331765 | 0.261358415 | negative | 0.334279919 | disease |
| HOMOCYSTEINE THIOLACTONE | 0.023151385 | 0.223264506 | negative | 0.524280452 | disease |
| INDOLEACRYLICACID | 0.02659894 | 0.242570771 | negative | 0.507529567 | disease |
| INDOXYL-BETA-D-GLUCURONIDE | 0.047788037 | 0.312360047 | negative | 0.394858541 | disease |
| INOSINE | 0.035356327 | 0.270316604 | negative | 0.649087068 | disease |
| L-ASCORBIC ACID 2-SULFATE | 0.005099016 | 0.135424411 | negative | 0.384486633 | disease |
| L-ASPARTIC ACID | 0.006011795 | 0.14010822 | negative | -0.29222253 | normal |
| LANTHIONINE KETIMINE | 0.006462808 | 0.140522812 | negative | 0.853786719 | disease |
| LIMONENE-1_2-DIOL | 0.030481244 | 0.253205581 | negative | -0.386494819 | normal |
| LYSOPHOSPHATIDYLCHOLINE 14:1(9Z)/0:0 | 0.015030301 | 0.191067005 | negative | -1.66231432 | normal |
| LYSOPHOSPHATIDYLETHANOLAMINE (22:6(4Z,7Z,10Z,13Z,16Z,19Z)/0:0) | 0.010388989 | 0.158479305 | negative | 1.649135766 | disease |
| METHIMAZOLE | 0.002150793 | 0.087656981 | negative | 1.661385123 | disease |
| METHYL PHTHALYL ETHYL GLYCOLATE | 0.010750661 | 0.161067943 | negative | 0.371753172 | disease |
| MFCD00025555 | 0.040881743 | 0.285208513 | negative | 0.227825337 | disease |
| MFCD00059633 | 0.015279084 | 0.191330614 | negative | -0.335659967 | normal |
| MOMILACTONEA | 0.022440239 | 0.22149836 | negative | 0.126163942 | disease |
| N-ACETYL-L-CARNOSINE | 0.047788037 | 0.312360047 | negative | 0.351415576 | disease |
| N-ACETYL-L-METHIONINE | 0.003371461 | 0.113146226 | negative | 0.296374857 | disease |
| N-ACETYLGLUCOSAMINITOL | 0.01712434 | 0.202356642 | negative | 0.336322098 | disease |
| N2-(D-1-CARBOXYETHYL)-L-LYSINE | 0.00198552 | 0.087656981 | negative | 0.411976343 | disease |
| NONADECANOICACID | 0.000549273 | 0.068661586 | negative | -1.272013907 | normal |
| NP-021038 | 0.00030588 | 0.051326741 | negative | 0.586400451 | disease |
| NP-021797 | 0.005388319 | 0.135424411 | negative | -0.643131379 | normal |
| O-PHOSPHORYLETHANOLAMINE | 0.036407361 | 0.270316604 | negative | 0.266593228 | disease |
| OLEOYL-L-Α-LYSOPHOSPHATIDIC ACID | 0.011123485 | 0.163729888 | negative | -0.948142562 | normal |
| RG5927903 | 0.020419545 | 0.214149982 | negative | 0.690988652 | disease |
| TCPY | 0.049833476 | 0.314363052 | negative | -0.559103234 | normal |
| THALIDOMIDE | 0.004313386 | 0.129247542 | negative | -0.471108055 | normal |
| THEOPHYLLINE | 0.035878562 | 0.270316604 | negative | -1.414759344 | normal |
| TRIDECYLIC ACID | 0.020419545 | 0.214149982 | negative | -0.653092891 | normal |
| UF7990000 | 0.037484906 | 0.273476834 | negative | 0.206351481 | disease |
| YQ2978400 | 0.007069263 | 0.144661261 | negative | -0.564013585 | normal |
| (+-)-ALBENDAZOLE SULFOXIDE | 0.004647907 | 0.131409 | positive | -0.51689376 | normal |
| (+/-)-CAMPHORIC ACID | 0.038589472 | 0.281721261 | positive | 0.669830657 | disease |
| (+/-)5(6)-EET | 0.032357239 | 0.255341938 | positive | -0.510341276 | normal |
| (1-RIBOSYLIMIDAZOLE)-4-ACETATE | 0.001293604 | 0.071841228 | positive | 0.233391806 | disease |
| (1S,3R,5Z,7E,9XI,20S)-20-(3-METHYLBUTOXY)-9,10-SECOPREGNA-5,7,10-TRIENE-1,3-DIOL | 0.015279084 | 0.1841781 | positive | 0.349269085 | disease |
| (1S,4S,5R,9R,13S)-5,9-DIMETHYL-14-METHYLIDENETETRACYCLO[11.2.1.0ÂA,ÂAÂ°.0Â´,ÂA]HEXADEC-10-ENE-5-CARBOXYLIC ACID | 0.049143655 | 0.323806707 | positive | 0.260663975 | disease |
| (2E_6E)-(10R_11S)-10_11-EPOXY-3_7_11-TRIMETHYLTRIDECA-2_6-DIENOICACID | 0.006580216 | 0.151743192 | positive | -0.415968641 | normal |
| (2R,5R,6R)-3-[(1E,3E)-HEPTA-1,3-DIEN-1-YL]-5,6-DIHYDROXY-2-(HYDROXYMETHYL)CYCLOHEXAN-1-ONE | 0.041472513 | 0.293135264 | positive | -0.101171287 | normal |
| (2R_4S)-2_4-DIAMINOPENTANOATE | 0.032841407 | 0.255341938 | positive | 0.144085644 | disease |
| (2S)-3-(BETA-D-GALACTOPYRANOSYLOXY)-2-[(9Z,12Z,15Z)-9,12,15-OCTADECATRIENOYLOXY]PROPYL (5Z,8Z,11Z,14Z,17Z)-5,8,11,14,17-ICOSAPENTAENOATE | 0.014543418 | 0.180920114 | positive | -0.275039457 | normal |
| (3,4-DIMETHOXYPHENYL)ACETIC ACID | 0.002569745 | 0.105156681 | positive | 0.256467904 | disease |
| (3E)-6-[16-(HEXOPYRANOSYLOXY)-19-METHOXY-5,9,17,17-TETRAMETHYL-18-OXAPENTACYCLO[10.5.2.0~1,13~.0~4,12~.0~5,9~]NONADEC-2-EN-8-YL]-2-METHYL-3-HEPTEN-2-YL HEXOPYRANOSIDE | 0.020744884 | 0.212225622 | positive | 0.648205686 | disease |
| (3R,4R)-4-HYDROXY-3-({4-[2-(4-MORPHOLINYL)ETHOXY]BENZOYL}AMINO)-N-PHENYL-1-AZEPANECARBOXAMIDE | 0.032357239 | 0.255341938 | positive | 0.3921495 | disease |
| (3S,4S,5R)-5-[6-(1,3-DIOXAN-2-YL)HEXYL]-4-[(1Z)-1-OCTEN-1-YL]-2,3-DIPHENYL-1,2-OXAZOLIDINE | 0.036407361 | 0.273494913 | positive | -0.532808044 | normal |
| (4S,5S,8S,10R)-4,5,8-TRIHYDROXY-10-METHYL-3,4,5,8,9,10-HEXAHYDRO-2H-OXECIN-2-ONE | 0.011314168 | 0.159941196 | positive | 0.102302625 | disease |
| (5Ξ,9Ξ,16Ξ)-17-HYDROXYKAURAN-19-OIC ACID | 0.025015449 | 0.22747967 | positive | -0.3570206 | normal |
| (9Z,13Z,15Z)-14,18-DIHYDROXY-12-OXO-9,13,15-OCTADECATRIENOIC ACID | 0.020744884 | 0.212225622 | positive | -0.658156593 | normal |
| [FA(18:3)]13S-HYDROPEROXY-9Z_11E_14Z-OCTADECATRIENOICACID | 0.012311783 | 0.170935915 | positive | 0.573091208 | disease |
| [SIMILAR TO: ETHYLENEDIAMINETETRAACETIC ACID (EDTA); ΔMASS: -0.0228 DA] | 0.003182611 | 0.111165698 | positive | 0.27865268 | disease |
| [SIMILAR TO: L-(+)-CITRULLINE; ΔMASS: 173.9561 DA] | 0.039721577 | 0.287288613 | positive | -0.085680073 | normal |
| [SIMILAR TO: L-GLUTAMIC ACID; ΔMASS: -0.0565 DA] | 0.035356327 | 0.268190675 | positive | 0.174099465 | disease |
| [SIMILAR TO: NP-018716; ΔMASS: 31.0246 DA] | 0.027422651 | 0.235592391 | positive | -0.308508181 | normal |
| [SIMILAR TO: NP-021797; ΔMASS: 59.0198 DA] | 0.02869945 | 0.242541545 | positive | -0.414104925 | normal |
| [ST(2:0)]5ALPHA-ANDROSTANE-3_17-DIONE | 0.007325821 | 0.151743192 | positive | 1.418136916 | disease |
| 1-(4-METHYL-1-PIPERAZINYL)ETHANONE | 0.009531746 | 0.152802727 | positive | 0.31245172 | disease |
| 1-(6-AMINO-9H-PURIN-9-YL)-1-DEOXY-Î²-D-RIBOFURANURONAMIDE | 0.009206903 | 0.151743192 | positive | 0.250203465 | disease |
| 1-(BETA-D-RIBOFURANOSYL)-1,2-DIHYDROPYRIMIDINE | 8.02623E-05 | 0.013780516 | positive | 0.332835618 | disease |
| 1-[(8Z,11Z,14Z)-ICOSATRIENOYL]-SN-GLYCERO-3-PHOSPHOCHOLINE | 0.006944034 | 0.151743192 | positive | 0.309141406 | disease |
| 1-METHYLGUANINE | 0.005005748 | 0.13656032 | positive | 0.252869299 | disease |
| 1-METHYLHISTIDINE | 0.007590701 | 0.151743192 | positive | 0.27493644 | disease |
| 1-PHENYL-3-METHYL-5-PYRAZOLONE | 0.04581363 | 0.309739975 | positive | 0.198936318 | disease |
| 1-VINYL-2-PYRROLIDONE | 0.005903567 | 0.151743192 | positive | 1.361149114 | disease |
| 1,2,3,4-TETRAHYDRO-Î²-CARBOLINE-3-CARBOXYLIC ACID | 0.01740331 | 0.197533917 | positive | 0.392233447 | disease |
| 1,4-DIHYDRO-4-IMINO-1-Î²-D-RIBOFURANOSYL-3-PYRIDINECARBOXYLIC ACID | 0.003244462 | 0.111165698 | positive | 0.258127748 | disease |
| 1,6-HEXANEDIOL DIGLYCIDYL ETHER | 0.004477817 | 0.131409 | positive | 0.324842388 | disease |
| 1_2-BENZOQUINONE | 8.17784E-11 | 1.27165E-07 | positive | 0.431105023 | disease |
| 10-SULFANYLDECYL METHACRYLATE | 0.04581363 | 0.309739975 | positive | -0.340138168 | normal |
| 10_16-DIHYDROXYHEXADECANOICACID | 0.000221097 | 0.023591827 | positive | -0.762815295 | normal |
| 13,14-DIHYDRO-15-KETO PROSTAGLANDIN A2 | 0.040881743 | 0.290279041 | positive | 0.392045465 | disease |
| 14(S)-HDHA | 0.013611195 | 0.176378407 | positive | 0.190346224 | disease |
| 1726111 | 0.045170957 | 0.309739975 | positive | -0.091928432 | normal |
| 1750713 | 0.003436643 | 0.111165698 | positive | 0.187657128 | disease |
| 2-(2-ETHOXYETHOXY)ETHANOL | 0.018264267 | 0.202863822 | positive | 0.606286711 | disease |
| 2-(DIETHOXYMETHYL)FURAN | 0.004914026 | 0.136451958 | positive | 0.460580579 | disease |
| 2-ETHYL-4,5-DIMETHYLTHIAZOLE | 0.008738153 | 0.151743192 | positive | 0.305705831 | disease |
| 2-HEXENOYLCARNITINE | 0.003244462 | 0.111165698 | positive | 0.503495287 | disease |
| 2-MERCAPTOETHANOL | 0.012947075 | 0.173557769 | positive | 0.334598395 | disease |
| 2-METHYL-6-OXOHEPTA-2_4-DIENAL | 0.020098666 | 0.211171796 | positive | 0.11876101 | disease |
| 2-OXO-2H-CHROMENE-3-CARBOXYLIC ACID | 0.011123485 | 0.159941196 | positive | 0.650307899 | disease |
| 2-OXOVALERICACID | 1.03886E-06 | 0.000538477 | positive | 0.457842971 | disease |
| 2,2,6,6-TETRAMETHYL-4-PIPERIDINOL | 0.009206903 | 0.151743192 | positive | 0.311332424 | disease |
| 2,5,7,8-TETRAMETHYL-2-[2-(PHENYLSULFANYL)ETHYL]-3,4-DIHYDRO-2H-CHROMEN-6-YL ACETATE | 0.027842696 | 0.237886769 | positive | -0.62619122 | normal |
| 2_5-DIOXOPIPERAZINE | 2.16459E-05 | 0.00591555 | positive | 0.818437875 | disease |
| 2_7-ANHYDRO-ALPHA-N-ACETYLNEURAMINICACID | 0.007196516 | 0.151743192 | positive | 0.447756939 | disease |
| 2475675 | 0.003371461 | 0.111165698 | positive | -0.44125573 | normal |
| 3-[(E)-2-[(1R,4AS,5R,6R,8AR)-6-HYDROXY-5-(HYDROXYMETHYL)-5,8A-DIMETHYL-2-METHYLIDENE-DECAHYDRONAPHTHALEN-1-YL]ETHENYL]-2,5-DIHYDROFURAN-2-ONE | 0.013386552 | 0.174925114 | positive | 0.584793717 | disease |
| 3-{[(CARBOXYCARBONYL)CARBAMOYL]AMINO}ALANINE | 0.015030301 | 0.184032422 | positive | 0.685380565 | disease |
| 3-DEHYDROCARNITINE | 0.000170537 | 0.020398802 | positive | 0.80368044 | disease |
| 3-DEHYDROXYCARNITINE | 0.02619516 | 0.233679956 | positive | 0.284151336 | disease |
| 3-HYDROXY-CIS-5-OCTENOYLCARNITINE | 0.002470668 | 0.105156681 | positive | 0.189672589 | disease |
| 3-HYDROXYCYCLOHEXANONE | 0.000640562 | 0.045276056 | positive | 0.234193704 | disease |
| 3-INDOLEBUTYRICACID | 0.022793402 | 0.222916609 | positive | 0.294268778 | disease |
| 3-ISOPROPYL-2,5-PIPERAZINEDIONE | 0.002282923 | 0.105156681 | positive | 0.216923922 | disease |
| 3-METHYL-5-OXO-5-(4-TOLUIDINO)PENTANOIC ACID | 0.02659894 | 0.233679956 | positive | 0.224903388 | disease |
| 3-METHYL-5-PHENYL-2-CYCLOHEXEN-1-ONE | 0.027422651 | 0.235592391 | positive | 0.244827853 | disease |
| 3-PYRIDINOL | 1.19106E-07 | 9.26053E-05 | positive | 0.389024158 | disease |
| 3,5-DIOXOOCTANEDIOIC ACID | 0.049143655 | 0.323806707 | positive | -0.791229801 | normal |
| 3_4-DIHYDROXYMANDELALDEHYDE | 8.64572E-05 | 0.013780516 | positive | 0.551331909 | disease |
| 4-COUMARYLALCOHOL | 0.034840592 | 0.268190675 | positive | 0.460100792 | disease |
| 4-HYDROXY-4-METHYL-2-PENTANONE | 0.019782196 | 0.210693939 | positive | 0.637759334 | disease |
| 4-SULFANYLDIHYDRO-3(2H)-FURANONE | 0.009206903 | 0.151743192 | positive | 0.393818836 | disease |
| 4-TERT-BUTYLPHENYL SALICYLATE | 0.032841407 | 0.255341938 | positive | -0.069060719 | normal |
| 4A-HYDROXYTETRAHYDROBIOPTERIN | 0.003436643 | 0.111165698 | positive | 0.775011519 | disease |
| 5'-METHYLTHIOADENOSINE | 8.02623E-05 | 0.013780516 | positive | 0.399741433 | disease |
| 5-(4-CARBOXY-3-METHYLBUTYL)-5,6,8A-TRIMETHYL-3-OXO-3,4,4A,5,6,7,8,8A-OCTAHYDRONAPHTHALENE-1-CARBOXYLIC ACID | 0.008004135 | 0.151743192 | positive | 0.566185177 | disease |
| 5-(HYDROXYMETHYL)-4-METHOXY-2,5-DIHYDROFURAN-2-ONE | 0.019162287 | 0.208373119 | positive | 0.155536408 | disease |
| 5-AMINO-1-(2-CYANOETHYL)-1H-PYRAZOLE-4-CARBONITRILE | 0.002945806 | 0.111165698 | positive | 0.62320103 | disease |
| 5-HYDROXYCONIFERYLALCOHOL | 0.002108332 | 0.105156681 | positive | 0.368964047 | disease |
| 609975 | 0.02869945 | 0.242541545 | positive | 0.256808399 | disease |
| 7-METHYLGUANINE | 0.006699538 | 0.151743192 | positive | 0.213261712 | disease |
| 8-OXOCOFORMYCIN | 2.28253E-05 | 0.00591555 | positive | 0.449111073 | disease |
| 8561 | 0.007864136 | 0.151743192 | positive | 0.382012529 | disease |
| 9-ALLYL-2-PHENYL-9H-IMIDAZO[1,2-A]BENZIMIDAZOLE | 0.042070497 | 0.296016392 | positive | 0.093348691 | disease |
| ACETONECYANOHYDRIN | 0.019470086 | 0.210249889 | positive | 0.184267978 | disease |
| ACETYLCADAVERINE | 0.003182611 | 0.111165698 | positive | 0.420674077 | disease |
| ALLYLCYSTEINE | 0.038033779 | 0.280296332 | positive | 0.655644576 | disease |
| AMPA | 0.02659894 | 0.233679956 | positive | -0.081771846 | normal |
| ANISOLE | 0.031407227 | 0.254216178 | positive | 0.181437288 | disease |
| ANKORINE | 0.009368068 | 0.151743192 | positive | 0.374703346 | disease |
| APRONALIDE | 0.009048217 | 0.151743192 | positive | 0.29447039 | disease |
| ARENEDIOL | 0.011314168 | 0.159941196 | positive | 0.320020824 | disease |
| ASPARTYL-L-PROLINE | 0.004647907 | 0.131409 | positive | 0.568081796 | disease |
| ASPARTYL-PHENYLALANINE | 0.02463257 | 0.22747967 | positive | 0.523533351 | disease |
| BENZOPHENONE | 0.010038184 | 0.153033101 | positive | 0.220218734 | disease |
| BUTYL ISOTHIOCYANATE | 0.002282923 | 0.105156681 | positive | 0.743035765 | disease |
| CAPROLACTAM | 0.007457206 | 0.151743192 | positive | 0.36995355 | disease |
| CAPRYLIC DIETHANOLAMIDE | 0.019162287 | 0.208373119 | positive | -0.117232673 | normal |
| CHOLINE | 0.007457206 | 0.151743192 | positive | 0.185146838 | disease |
| CYCLOHEXYLAMINE | 0.030027113 | 0.251033121 | positive | 0.329607029 | disease |
| CYTIDINE | 0.020744884 | 0.212225622 | positive | 0.351633581 | disease |
| D-(+)-PROLINE | 0.007726335 | 0.151743192 | positive | 0.199609678 | disease |
| DELTA-GUANIDINOVALERICACID | 0.018264267 | 0.202863822 | positive | 0.362895822 | disease |
| DETHIOBIOTIN | 0.000964331 | 0.059981363 | positive | 0.537694384 | disease |
| DEZAGUANINE | 8.86207E-05 | 0.013780516 | positive | 0.453540304 | disease |
| DI-ISOAMYLTHIOMALATE | 0.02174816 | 0.216784544 | positive | -0.52918392 | normal |
| DIBUTYL MALATE | 0.00114149 | 0.065741387 | positive | -0.749008311 | normal |
| DIETHYL PHOSPHATE | 0.014305232 | 0.180850697 | positive | 0.16375168 | disease |
| DIETHYLENE GLYCOL | 0.01252041 | 0.172294131 | positive | 0.408430131 | disease |
| DIETHYLENE GLYCOL N-BUTYL ETHER | 0.016310835 | 0.192146581 | positive | 0.591616837 | disease |
| DIPROPYLENE GLYCOL DIMETHYL ETHER | 0.025015449 | 0.22747967 | positive | 0.364333348 | disease |
| DL-STACHYDRINE | 0.039721577 | 0.287288613 | positive | 0.853966672 | disease |
| DODECYLETHANOL AMIDE | 0.010212246 | 0.15385598 | positive | 0.087291806 | disease |
| EDDA | 0.011123485 | 0.159941196 | positive | -0.152981179 | normal |
| EPIRIZOLE | 0.007457206 | 0.151743192 | positive | 0.317315178 | disease |
| ETHOPABATE | 0.000964331 | 0.059981363 | positive | 0.427502145 | disease |
| ETHYL N-[(2-ISOPROPYL-5-METHYLCYCLOHEXYL)CARBONYL]GLYCINATE | 0.00623363 | 0.151743192 | positive | 0.31293157 | disease |
| FURAN FATTY ACID F6 | 0.02619516 | 0.233679956 | positive | -0.519777293 | normal |
| GAMMA-GLUTAMYL-GAMMA-AMINOBUTYRAYE | 0.025015449 | 0.22747967 | positive | 0.47979583 | disease |
| GINGERDIONE | 0.005193852 | 0.139248955 | positive | -0.133587102 | normal |
| GLU-GLY | 0.000327633 | 0.028303878 | positive | -0.109475549 | normal |
| GUANINE | 0.030941262 | 0.254216178 | positive | 0.463791923 | disease |
| IMIDAZOL-5-YL-PYRUVATE | 0.000358894 | 0.029372642 | positive | 0.318745932 | disease |
| IN00485 | 0.031879199 | 0.254216178 | positive | -0.384282623 | normal |
| INDOLE;1-BENZAZOLE | 0.023882019 | 0.22506994 | positive | 0.205667333 | disease |
| INDOLINE | 0.00100606 | 0.060170155 | positive | 0.326318108 | disease |
| INDOLYLMETHYL-DESULFOGLUCOSINOLATE | 0.02463257 | 0.22747967 | positive | 0.397755331 | disease |
| INOSINE | 0.007196516 | 0.151743192 | positive | 0.861262462 | disease |
| INOSINE-5'-MONOPHOSPHATE (IMP) | 2.5318E-06 | 0.000984239 | positive | 0.507835665 | disease |
| L-GAMMA-GLUTAMYL-L-LEUCINE | 0.009368068 | 0.151743192 | positive | 0.252068223 | disease |
| L-INDOSPICINE | 0.047122101 | 0.315839944 | positive | 0.374379013 | disease |
| L-ISOLEUCYL-L-ALANYL-D-ARGININE | 0.009206903 | 0.151743192 | positive | 0.068248019 | disease |
| L-N2-(2-CARBOXYETHYL)ARGININE | 0.007864136 | 0.151743192 | positive | 0.396327311 | disease |
| L-NORLEUCINE | 0.000640562 | 0.045276056 | positive | 0.234625211 | disease |
| L(-)-CARNITINE | 0.031407227 | 0.254216178 | positive | 0.167268033 | disease |
| MANIDIPINE | 0.031407227 | 0.254216178 | positive | -0.477017738 | normal |
| METHIMAZOLE | 0.000272605 | 0.024935319 | positive | 2.149002326 | disease |
| METHYL 2,3-DIHYDRO-3-HYDROXY-2-OXO-1H-INDOLE-3-ACETATE | 0.031879199 | 0.254216178 | positive | 0.341984245 | disease |
| MFCD00025555 | 0.010388989 | 0.15385598 | positive | 0.281729017 | disease |
| MFCD00037215 | 0.002889174 | 0.111165698 | positive | 0.50071281 | disease |
| MFCD00210473 | 0.00986677 | 0.153033101 | positive | 0.303508414 | disease |
| MORPHINE-D3 | 0.02351424 | 0.222955137 | positive | 0.579139258 | disease |
| N-[(4-METHOXY-1-BENZOFURAN-5-YL)CARBONYL]GLYCINE | 0.047122101 | 0.315839944 | positive | -0.544216868 | normal |
| N-{(3S,5S)-1-METHYL-5-[3-(4-METHYLPHENYL)-1,2,4-OXADIAZOL-5-YL]-3-PYRROLIDINYL}-2-PHENYLACETAMIDE | 0.002375098 | 0.105156681 | positive | -0.429868293 | normal |
| N-ACETYL-ALA-ALA-ALA-METHYLESTER | 0.00858671 | 0.151743192 | positive | -0.151394555 | normal |
| N-ACETYL-L-METHIONINE | 0.000131004 | 0.018519247 | positive | 0.396608329 | disease |
| N-ACETYL-L-PHENYLALANINE | 0.019162287 | 0.208373119 | positive | 0.285495606 | disease |
| N-AMIDINO-L-ASPARTATE | 0.000830573 | 0.056153927 | positive | 0.243848444 | disease |
| N-ETHYLMALEIMIDE-S-GLUTATHIONE | 0.038033779 | 0.280296332 | positive | 0.291614068 | disease |
| N-METHYLDODECANAMIDE | 0.008437618 | 0.151743192 | positive | -0.135350098 | normal |
| N(3)-(4-METHOXYFUMAROYL)-2,3-DIAMINOPROPIONIC ACID | 0.035356327 | 0.268190675 | positive | -0.312692649 | normal |
| N,N′-DIETHYLTHIOUREA | 0.010038184 | 0.153033101 | positive | 0.784858135 | disease |
| N~2~-(3-HYDROXYHEXADECANOYL)-L-ORNITHINE | 0.007069263 | 0.151743192 | positive | 0.12341313 | disease |
| N~2~-STEAROYL-L-GLUTAMINE | 0.023151385 | 0.222955137 | positive | 0.193824963 | disease |
| N2-(D-1-CARBOXYETHYL)-L-LYSINE | 0.003502971 | 0.111165698 | positive | 0.424059246 | disease |
| N2-DIMETHYLGUANOSINE | 0.009048217 | 0.151743192 | positive | 0.276396896 | disease |
| N6-ACETYL-L-LYSINE | 0.000155015 | 0.02008738 | positive | 0.248830692 | disease |
| N6-THREONYLCARBAMOYLADENOSINE | 0.005487998 | 0.144641293 | positive | 0.276870749 | disease |
| NICOTIANAMINE | 0.013839162 | 0.176392603 | positive | 0.433645062 | disease |
| NICOTINURICACID | 0.031879199 | 0.254216178 | positive | 0.371172737 | disease |
| NP-000919 | 0.021074732 | 0.214190904 | positive | 0.63836819 | disease |
| NP-012972 | 0.016849302 | 0.195527347 | positive | -0.415925113 | normal |
| NP-013210 | 0.013386552 | 0.174925114 | positive | 0.215681117 | disease |
| NP-014175 | 0.00300345 | 0.111165698 | positive | 0.297303613 | disease |
| NP-015090 | 0.045170957 | 0.309739975 | positive | -0.143224172 | normal |
| NP-019992 | 0.007864136 | 0.151743192 | positive | 0.377405697 | disease |
| NP-020156 | 0.000242745 | 0.023591827 | positive | 0.639717488 | disease |
| NP-022451 | 0.011704263 | 0.163965119 | positive | 0.387428785 | disease |
| NPYR | 0.02351424 | 0.222955137 | positive | 0.172098007 | disease |
| O-(17-CARBOXYHEPTADECANOYL)CARNITINE | 0.044535902 | 0.309739975 | positive | 0.273440215 | disease |
| OGLUFANIDE | 0.038033779 | 0.280296332 | positive | 0.215781114 | disease |
| PEG N7 | 0.040881743 | 0.290279041 | positive | 0.254517902 | disease |
| PHENYLGLYOXYLIC ACID | 0.013839162 | 0.176392603 | positive | 0.476625765 | disease |
| PIPECOLIC ACID | 0.04581363 | 0.309739975 | positive | 0.647806386 | disease |
| PIRACETAM | 0.027422651 | 0.235592391 | positive | 0.296510792 | disease |
| PREGNA-4_9(11)-DIENE-3_20-DIONE | 0.045170957 | 0.309739975 | positive | 0.258645592 | disease |
| PROPOFOL | 0.043908396 | 0.307556555 | positive | -0.37376931 | normal |
| RABEPRAZOLE | 0.027008085 | 0.235592391 | positive | 0.544135079 | disease |
| RICININE | 0.000237154 | 0.023591827 | positive | 0.571837463 | disease |
| S-ADENOSYLHOMOCYSTEINE(ADOHCY) | 0.000480828 | 0.037384362 | positive | 0.433643179 | disease |
| S-METHYL-1-THIO-D-GLYCERATE | 0.01273216 | 0.173557769 | positive | 0.289664966 | disease |
| S-METHYL-L-CYSTEINE-S-OXIDE | 0.007069263 | 0.151743192 | positive | 0.591997916 | disease |
| STYRENE | 0.035356327 | 0.268190675 | positive | 0.246738708 | disease |
| SULAR | 0.002066641 | 0.105156681 | positive | 0.504836672 | disease |
| SYMMETRICDIMETHYLARGININE | 0.002519761 | 0.105156681 | positive | 0.230168543 | disease |
| TETRAACETYLETHYLENEDIAMINE | 0.004647907 | 0.131409 | positive | 0.40412686 | disease |
| THIOPHENE | 0.033828373 | 0.261707067 | positive | 0.412496712 | disease |
| TOLUENE | 0.025796689 | 0.233220067 | positive | 0.232225513 | disease |
| TRANEXAMIC ACID | 0.020744884 | 0.212225622 | positive | 0.361871949 | disease |
| UROCANIC ACID | 0.02174816 | 0.216784544 | positive | 0.253271654 | disease |
| VALPROMIDE | 0.007325821 | 0.151743192 | positive | -0.448292964 | normal |
| VALPROMIDE | 0.00378009 | 0.115255673 | positive | 0.560484863 | disease |
| VISNAGIN | 0.030027113 | 0.251033121 | positive | -0.227094537 | normal |
| WELKSTOFF | 0.002025708 | 0.105156681 | positive | -0.66031815 | normal |
| XANTHOSINE | 0.022793402 | 0.222916609 | positive | 0.316184452 | disease |
| YK6000000 | 0.008437618 | 0.151743192 | positive | 0.351228118 | disease |
| Α-ASPARTYLPHENYLALANINE | 0.015279084 | 0.1841781 | positive | 0.386394504 | disease |
| Β-ALANINE | 0.002519761 | 0.105156681 | positive | 0.236373014 | disease |
| Υ-L-GLUTAMYL-L-GLUTAMIC ACID | 0.036407361 | 0.273494913 | positive | 0.230910094 | disease |
| Abbreviations: DEM, differentially expressed metabolite; FDR, false discovery rate; logFC, logarithm of Fold Change. | | | | | |

| **Table S2. Results of DEMs in untargeted metabolomics (Non-DON Group vs. DON Group)** | | | | | |
| --- | --- | --- | --- | --- | --- |
| DEM | *P* | FDR | Pattern | logFC | sig |
| [SIMILAR TO: ALLOPURINOL; ΔMASS: 158.0206 DA] | 0.040879527 | 0.97009216 | negative | -0.879990678 | Non-DON |
| [SIMILAR TO: N-{[2-(2-THIENYL)-1,3-THIAZOL-4-YL]METHYL}BENZAMIDE; ΔMASS: 0.0014 DA] | 0.026010316 | 0.97009216 | negative | -0.071796598 | Non-DON |
| [SIMILAR TO: N-BUTYLBENZENESULFONAMIDE; ΔMASS: 0.0909 DA] | 0.040879527 | 0.97009216 | negative | -0.283408436 | Non-DON |
| 2,3-O-ISOPROPYLIDENE-L-THREITOL | 0.013981869 | 0.97009216 | negative | -0.576304072 | Non-DON |
| 4-VINYLPHENOL SULFATE | 0.046109828 | 0.97009216 | negative | -0.489495712 | Non-DON |
| 5-HYDROXYCONIFERYLALCOHOL | 0.019976748 | 0.97009216 | negative | -0.781403243 | Non-DON |
| ANDROSTERONE GLUCURONIDE | 0.008314197 | 0.97009216 | negative | -0.730478943 | Non-DON |
| DL-MANDELIC ACID | 0.04722086 | 0.97009216 | negative | -0.810780715 | Non-DON |
| ETHYL 4-(METHYLSULFONYL)BENZOATE | 0.028103417 | 0.97009216 | negative | -0.906250073 | Non-DON |
| ETHYL PARABEN | 0.008564541 | 0.97009216 | negative | -1.040532607 | Non-DON |
| GUAIACOL SULFATE | 0.038932921 | 0.97009216 | negative | -0.829339232 | Non-DON |
| HYPOXANTHINE | 0.04290799 | 0.97009216 | negative | -0.533100795 | Non-DON |
| INDOLE-3-ACETIC ACID | 0.021074955 | 0.97009216 | negative | -0.384801767 | Non-DON |
| LYSOPHOSPHATIDYLETHANOLAMINE (22:6(4Z,7Z,10Z,13Z,16Z,19Z)/0:0) | 0.017447105 | 0.97009216 | negative | -1.41176063 | Non-DON |
| METHYL SALICYLATE | 0.008821602 | 0.97009216 | negative | -0.628191302 | Non-DON |
| MFCD00040496 | 0.049510547 | 0.97009216 | negative | 0.650382363 | DON |
| PENTADECANOIC ACID | 0.014378878 | 0.97009216 | negative | 0.58172154 | DON |
| PYRUVIC ACID | 0.022820824 | 0.97009216 | negative | 0.503038999 | DON |
| TAURINE | 0.033558994 | 0.97009216 | negative | -0.312753354 | Non-DON |
| (1R)-1,2-BIS[(7-{(2R,3R)-3-[(4Z,6Z)-4,6-NONADIEN-1-YL]-2-OXIRANYL}-4,6-HEPTADIYNOYL)OXY]ETHYL MYRISTATE | 0.029578119 | 0.944772639 | positive | 0.386971745 | DON |
| (2BETA,3ALPHA,22S)-3,20,22-TRIHYDROXY-6-OXOCHOLESTA-4,7-DIEN-2-YL ACETATE | 0.040879527 | 0.944772639 | positive | 0.228695317 | DON |
| (2E,6E)-N,3,7,11-TETRAMETHYL-2,6,10-DODECATRIEN-1-AMINE | 0.04722086 | 0.944772639 | positive | -0.264861101 | Non-DON |
| (2R,3R,4S,5S,6R)-2-{[(2E,6R)-6-HYDROXY-2,6-DIMETHYLOCTA-2,7-DIEN-1-YL]OXY}-6-(HYDROXYMETHYL)OXANE-3,4,5-TRIOL | 0.027390135 | 0.944772639 | positive | -0.814629475 | Non-DON |
| (2S,3S,4S,5R,6R)-6-[3-(BENZOYLOXY)-2-HYDROXYPROPOXY]-3,4,5-TRIHYDROXYOXANE-2-CARBOXYLIC ACID | 0.048354321 | 0.944772639 | positive | -0.696085682 | Non-DON |
| (4R)-4-{(2E,6E)-9-[(2S)-3,3-DIMETHYL-2-OXIRANYL]-3,7-DIMETHYL-2,6-NONADIEN-1-YL}-7-HYDROXY-4,6-DIMETHYL-2-BENZOFURAN-1,5(3H,4H)-DIONE | 0.019976748 | 0.944772639 | positive | 0.324541456 | DON |
| (6Z)-OCTADECENOICACID | 0.021074955 | 0.944772639 | positive | 0.355847716 | DON |
| [SIMILAR TO: 18-Β-GLYCYRRHETINIC ACID; ΔMASS: -0.0382 DA] | 0.04722086 | 0.944772639 | positive | 0.245853128 | DON |
| [SIMILAR TO: L-GLUTAMIC ACID; ΔMASS: -0.0565 DA] | 0.006148344 | 0.944772639 | positive | -0.374000152 | Non-DON |
| 1-(4-METHYL-1-PIPERAZINYL)-2-[(3R,4S)-3-{[5-(PHENOXYMETHYL)-1,2-OXAZOL-3-YL]METHYL}-4-PIPERIDINYL]ETHANONE | 0.028832651 | 0.944772639 | positive | 0.439025085 | DON |
| 13-AMINOTRIDECANOIC ACID | 0.010213142 | 0.944772639 | positive | 0.084550841 | DON |
| 3-PYRIDINOL | 0.013981869 | 0.944772639 | positive | 0.138845417 | DON |
| 3A-HYDROXY-3-(HYDROXYMETHYL)-1,1,3,5-TETRAMETHYL-OCTAHYDRO-1H-INDENE-4-CARBOXYLIC ACID | 0.037065627 | 0.944772639 | positive | 0.426215785 | DON |
| 4-METHYLENE-2-OXOGLUTARATE | 0.031914774 | 0.944772639 | positive | -0.517799631 | Non-DON |
| 5-[(8Z)-8-HEPTADECEN-1-YL]-1,3-BENZENEDIOL | 0.046109828 | 0.944772639 | positive | -0.291050969 | Non-DON |
| 5-HYDROXYCONIFERALDEHYDE | 0.045020893 | 0.944772639 | positive | -0.567859555 | Non-DON |
| 6-(3-HYDROXYBUTAN-2-YL)-5-(HYDROXYMETHYL)-4-METHOXY-2H-PYRAN-2-ONE | 0.04290799 | 0.944772639 | positive | 0.147455489 | DON |
| 6-[8-HYDROXY-1-(HYDROXYMETHYL)OCTAHYDRO-2H-QUINOLIZIN-3-YL]-2-PIPERIDINONE | 0.025343233 | 0.944772639 | positive | 0.596313529 | DON |
| AC 45594 | 0.031118891 | 0.944772639 | positive | -0.118890106 | Non-DON |
| CALLYSTATIN A | 0.037989518 | 0.944772639 | positive | 0.721459061 | DON |
| CAY10401 | 0.037989518 | 0.944772639 | positive | 0.806321981 | DON |
| CYPRINOL | 0.028103417 | 0.944772639 | positive | 0.3809921 | DON |
| D-617 | 0.045020893 | 0.944772639 | positive | 0.296071203 | DON |
| DIDANOSINE | 0.04722086 | 0.944772639 | positive | 0.374687036 | DON |
| ETHYL 2-[3-CHLORO-5-(TRIFLUOROMETHYL)-2-PYRIDYL]-4-METHYL-1,3-THIAZOLE-5-CARBOXYLATE | 0.021074955 | 0.944772639 | positive | -0.143211224 | Non-DON |
| ETIOCHOLANOLONE | 0.023430064 | 0.944772639 | positive | -0.501392285 | Non-DON |
| EUGENOLMETHYLETHER | 0.031118891 | 0.944772639 | positive | 0.10844743 | DON |
| GUAIACOL SULFATE | 0.040879527 | 0.944772639 | positive | -0.2510235 | Non-DON |
| HYPOXANTHINE | 0.039896152 | 0.944772639 | positive | -0.611058181 | Non-DON |
| INDOLE-3-ACETIC ACID | 0.043953723 | 0.944772639 | positive | -0.365744329 | Non-DON |
| INOSINE | 0.040879527 | 0.944772639 | positive | -0.838926502 | Non-DON |
| L(-)-CARNITINE | 0.017447105 | 0.944772639 | positive | -0.250316475 | Non-DON |
| LAMTIDINE | 0.045020893 | 0.944772639 | positive | -0.422497231 | Non-DON |
| METHYL 3,4,5-TRIMETHOXYCINNAMATE | 0.022225395 | 0.944772639 | positive | -1.076869504 | Non-DON |
| METOMINOSTROBIN | 0.032728043 | 0.944772639 | positive | 0.536450645 | DON |
| MFCD00083078 | 0.049510547 | 0.944772639 | positive | -0.034404142 | Non-DON |
| MFCD28016586 | 0.011138922 | 0.944772639 | positive | 0.802730576 | DON |
| MIDAZOLAM | 0.041883366 | 0.944772639 | positive | -0.843916638 | Non-DON |
| N-(HENEICOSANOYL)-[(4E,6E)-TETRADECASPHINGADIENINE]-1-PHOSPHOETHANOLAMINE | 0.04722086 | 0.944772639 | positive | 0.34979576 | DON |
| N-ACETYLGLUTAMINE | 0.026010316 | 0.944772639 | positive | 0.263105887 | DON |
| N-HYDROXY-2-ACETAMIDOFLUORENE | 0.034407925 | 0.944772639 | positive | -0.625250194 | Non-DON |
| N-UNDECANOYLGLYCINE | 0.035275138 | 0.944772639 | positive | 0.3166737 | DON |
| NONYLPHENOXYACETIC ACID | 0.021074955 | 0.944772639 | positive | 0.356119969 | DON |
| QUINOLINE | 0.04722086 | 0.944772639 | positive | -0.330797572 | Non-DON |
| RABEPRAZOLE | 0.049510547 | 0.944772639 | positive | -0.341906732 | Non-DON |
| S-ADENOSYLHOMOCYSTEINE(ADOHCY) | 0.037065627 | 0.944772639 | positive | -0.214452736 | Non-DON |
| Abbreviations: DON, dysthyroid optic neuropathy; DEM, differentially expressed metabolite; FDR, False Discovery Rate; logFC, logarithm of Fold Change. | | | | | |

| **Table S3: Results of DEMs in targeted metabolomics (Normal Group vs. Disease Group)** | | | | |
| --- | --- | --- | --- | --- |
| DEM | *P* | FDR | logFC | sig |
| GLYCOLITHOCHOLIC ACID-3-SULFATE | 0.02319027 | 0.151957293 | -0.776765794 | normal |
| LITHOCHOLIC ACID （LCA） | 0.041013463 | 0.226941161 | -0.278746473 | normal |
| SEROTONIN | 0.007418933 | 0.065975507 | 0.860249398 | disease |
| 4Z,7Z,10Z,13Z,16Z,19Z-DOCOSAHEXAENOIC ACID (DHA) | 0.034852971 | 0.197236131 | -0.374854933 | normal |
| LIGNOCERIC ACID | 1.56911E-06 | 7.81416E-05 | 4.104561473 | disease |
| DIDECANOYLPHOSPHATIDYLCHOLINE | 0.00261171 | 0.029559807 | -0.242227611 | normal |
| THYROXINE | 0.020151669 | 0.143364734 | 1.470999245 | disease |
| MALTOTRIOSE | 0.001098776 | 0.016093831 | 1.004102417 | disease |
| GLYCINE | 0.00419097 | 0.041742065 | -0.245222261 | normal |
| CITRULLINE | 0.005161953 | 0.049435629 | -0.229845716 | normal |
| SHIKIMIC ACID | 0.004103614 | 0.041742065 | -0.592778316 | normal |
| ARGININE | 0.022406759 | 0.150791432 | -0.213322089 | normal |
| ASPARAGINE | 0.01715336 | 0.129429898 | -0.148347532 | normal |
| 3-PHOSPHOGLYCERATE | 0.001266658 | 0.017522097 | 0.302920253 | disease |
| ADENOSINE-5′-TRIPHOSPHATE | 1.75584E-07 | 1.56607E-05 | 1.113982341 | disease |
| ADENOSINE-5′-DIPHOSPHATE | 4.83519E-05 | 0.001203963 | 0.941538115 | disease |
| HISTIDINE | 0.000146588 | 0.002807729 | -0.144884212 | normal |
| CARNITINE | 0.007287497 | 0.065975507 | -0.219378395 | normal |
| CYSTINE | 7.8113E-05 | 0.001666592 | 0.29205284 | disease |
| PIPECOLIC ACID | 0.015949398 | 0.128109684 | -0.413404719 | normal |
| SERINE | 0.011403816 | 0.097915522 | -0.171382469 | normal |
| THREONINE | 0.001669824 | 0.02072049 | -0.236012119 | normal |
| TYROSINE | 0.01454937 | 0.120759772 | -0.106956066 | normal |
| GLUTAMIC ACID | 0.000618351 | 0.009623086 | 0.452023686 | disease |
| HOMOSERINE | 0.001669824 | 0.02072049 | -0.230224177 | normal |
| GLUTAMINE | 8.03177E-05 | 0.001666592 | -0.162075038 | normal |
| CITICOLINE | 1.35983E-05 | 0.000564331 | 1.411955813 | disease |
| 1-AMINOCYCLOPROPANECARBOXYLIC ACID | 0.000339099 | 0.005629049 | -0.270898137 | normal |
| DGTP | 5.84452E-08 | 1.45528E-05 | 1.141432964 | disease |
| 4-TRIMETHYLAMMONIOBUTANOIC ACID | 0.025743056 | 0.160250527 | -0.145875296 | normal |
| ACETYLCARNITINE | 0.016845124 | 0.129429898 | -0.152427098 | normal |
| DGDP | 3.6257E-05 | 0.001003111 | 0.989968252 | disease |
| TRIMETHYLLYSINE | 0.020884365 | 0.144450191 | -0.25264122 | normal |
| ADENOSINE 3',5'-DIPHOSPHATE | 1.78571E-05 | 0.000635202 | 1.008724912 | disease |
| N-ALPHA-ACETYLLYSINE | 0.018107949 | 0.132614095 | -0.191407258 | normal |
| 4-GUANIDINOBUTANOIC ACID | 0.003388673 | 0.03668607 | -0.168656747 | normal |
| ADP-RIBOSE | 4.26313E-07 | 2.6538E-05 | 1.256135948 | disease |
| ITP | 1.88684E-07 | 1.56607E-05 | 1.079707159 | disease |
| GLYCEROL 3-PHOSPHATE | 3.32297E-05 | 0.001003111 | -0.590380066 | normal |
| 2-PHOSPHOGLYCERIC ACID | 0.001747511 | 0.02072049 | 0.381855899 | disease |
| Abbreviations: DEM, differentially expressed metabolite; FDR, False Discovery Rate; logFC, logarithm of Fold Change. | | | | |

| **Table S4: Results of DEMs in targeted metabolomics (Non-DON Group vs. DON Group)** | | | | |
| --- | --- | --- | --- | --- |
| DEM | P | FDR | logFC | sig |
| GLYCOCHENODEOXYCHOLIC ACID (GCDCA) | 0.036551209 | 0.516537587 | 0.873830904 | DON |
| GLYCOURSODEOXYCHOLIC ACID (GUDCA) | 0.016492949 | 0.395627464 | 0.717562951 | DON |
| PALMITOYLETHANOLAMIDE | 0.003095676 | 0.256941135 | 0.846404344 | DON |
| GALLIC ACID | 0.00108949 | 0.135641549 | -1.839147559 | Non-DON |
| DIHOMO-GAMMA-LINOLENIC ACID | 0.045543472 | 0.532834196 | 0.27841598 | DON |
| ARACHIDONIC ACID | 0.012472647 | 0.395627464 | 0.337043212 | DON |
| THYROXINE | 0.017477518 | 0.395627464 | -1.939167025 | Non-DON |
| CAFFEINE | 0.022506988 | 0.451146117 | -1.696922205 | Non-DON |
| THEOPHYLLINE | 0.043651633 | 0.532834196 | -0.838028174 | Non-DON |
| AMINOADIPIC ACID | 0.031701034 | 0.516537587 | -0.24573675 | Non-DON |
| CITRAMALIC ACID | 0.013706134 | 0.395627464 | 0.204094158 | DON |
| CITRULLINE | 0.035857173 | 0.516537587 | 0.224455946 | DON |
| CITRIC ACID | 0.000128824 | 0.032077267 | 0.706796716 | DON |
| ISOCITRIC ACID | 0.049217617 | 0.532834196 | -0.232201299 | Non-DON |
| MALIC ACID | 0.023553813 | 0.451146117 | 0.246219985 | DON |
| 3-PHOSPHOGLYCERATE | 0.008451548 | 0.395627464 | -0.317032026 | Non-DON |
| ALLANTOIN | 0.049217617 | 0.532834196 | 0.385135735 | DON |
| TRANS-ACONITIC ACID | 0.0172615 | 0.395627464 | 0.271940925 | DON |
| N-ALPHA-ACETYLLYSINE | 0.011336707 | 0.395627464 | 0.246338078 | DON |
| ASYMMETRIC DIMETHYLARGININE | 0.008451548 | 0.395627464 | 0.254771044 | DON |
| DEOXYADENOSINE | 0.043385654 | 0.532834196 | 3.439997781 | DON |
| ITP | 0.037340067 | 0.516537587 | -0.629621407 | Non-DON |
| Abbreviations: DON, dysthyroid optic neuropathy; DEM, differentially expressed metabolite; FDR, False Discovery Rate; logFC, logarithm of Fold Change. | | | | |

| Table S5: Pathway enrichment analysis of untargeted metabolomics (Normal Group vs. Disease Group) | | | | | | | | |
| --- | --- | --- | --- | --- | --- | --- | --- | --- |
| Metabolism | HMDB | KEGG | Pathway | Total | Expected | Hits | Raw *P* | Sig |
| Urocanic acid | HMDB0000301 | C00785 | Histidine metabolism | 16 | 1.3 | 2 | 0.379 | up |
| L-Aspartic acid | HMDB0000191 | C00049 | Histidine metabolism | 16 | 1.3 | 2 | 0.379 | down |
| L-Aspartic acid | HMDB0000191 | C00049 | Pantothenate and CoA biosynthesis | 19 | 1.55 | 2 | 0.467 | down |
| Beta-Alanine | HMDB0000056 | C00099 | Pantothenate and CoA biosynthesis | 19 | 1.55 | 2 | 0.467 | up |
| L-Aspartic acid | HMDB0000191 | C00049 | beta-Alanine metabolism | 21 | 1.71 | 2 | 0.521 | down |
| Beta-Alanine | HMDB0000056 | C00099 | beta-Alanine metabolism | 21 | 1.71 | 2 | 0.521 | up |
| Arachidonic acid | HMDB0001043 | C00219 | Biosynthesis of unsaturated fatty acids | 36 | 2.93 | 3 | 0.573 | down |
| Docosahexaenoic acid | HMDB0002183 | C06429 | Biosynthesis of unsaturated fatty acids | 36 | 2.93 | 3 | 0.573 | down |
| Eicosapentaenoic acid | HMDB0001999 | C06428 | Biosynthesis of unsaturated fatty acids | 36 | 2.93 | 3 | 0.573 | down |
| L-Aspartic acid | HMDB0000191 | C00049 | Arginine biosynthesis | 14 | 1.14 | 1 | 0.697 | down |
| L-Aspartic acid | HMDB0000191 | C00049 | Nicotinate and nicotinamide metabolism | 14 | 1.14 | 1 | 0.697 | down |
| Glyceric acid | HMDB0000139 | C00258 | Glycerolipid metabolism | 16 | 1.3 | 1 | 0.745 | up |
| Glyceric acid | HMDB0000139 | C00258 | Glycine, serine and threonine metabolism | 33 | 2.69 | 2 | 0.766 | up |
| Choline | HMDB0000097 | C00114 | Glycine, serine and threonine metabolism | 33 | 2.69 | 2 | 0.766 | up |
| 5'-Methylthioadenosine | HMDB0001173 | C00170 | Cysteine and methionine metabolism | 33 | 2.69 | 2 | 0.766 | up |
| 2-Aminoacrylic acid | HMDB0003609 | C02218 | Cysteine and methionine metabolism | 33 | 2.69 | 2 | 0.766 | down |
| Inosinic acid | HMDB0000175 | C00130 | Purine metabolism | 64 | 5.21 | 4 | 0.782 | up |
| Xanthosine | HMDB0000299 | C01762 | Purine metabolism | 64 | 5.21 | 4 | 0.782 | up |
| Inosine | HMDB0000195 | C00294 | Purine metabolism | 64 | 5.21 | 4 | 0.782 | up |
| Guanine | HMDB0000132 | C00242 | Purine metabolism | 64 | 5.21 | 4 | 0.782 | up |
| Abbreviations: HMDB, Human Metabolome Database; KEGG, Kyoto Encyclopedia of Genes and Genomes. | | | | | | | | |

| **Table S6: Pathway enrichment analysis of untargeted metabolomics (Non-DON Group vs. DON Group)** | | | | | | | | |
| --- | --- | --- | --- | --- | --- | --- | --- | --- |
| Metabolism | HMDB | KEGG | Pathway | Total | Expected | Hits | Raw *P* | Sig |
| Taurine | HMDB0000251 | C00245 | Taurine and hypotaurine metabolism | 8 | 0.121 | 1 | 0.115 | down |
| Hypoxanthine | HMDB0000157 | C00262 | Purine metabolism | 64 | 0.967 | 2 | 0.252 | down |
| Inosine | HMDB0000195 | C00294 | Purine metabolism | 64 | 0.967 | 2 | 0.252 | down |
| Pyruvic acid | HMDB0000243 | C00022 | Citrate cycle (TCA cycle) | 20 | 0.302 | 1 | 0.264 | up |
| Pyruvic acid | HMDB0000243 | C00022 | Pyruvate metabolism | 22 | 0.332 | 1 | 0.286 | up |
| Pyruvic acid | HMDB0000243 | C00022 | Glycolysis / Gluconeogenesis | 26 | 0.393 | 1 | 0.329 | up |
| Pyruvic acid | HMDB0000243 | C00022 | Alanine, aspartate and glutamate metabolism | 28 | 0.423 | 1 | 0.35 | up |
| Etiocholanolone | HMDB0000490 | C04373 | Steroid hormone biosynthesis | 83 | 1.25 | 2 | 0.36 | down |
| Androsterone glucuronide | HMDB0002829 | C11135 | Steroid hormone biosynthesis | 83 | 1.25 | 2 | 0.36 | down |
| Pyruvic acid | HMDB0000243 | C00022 | Glyoxylate and dicarboxylate metabolism | 32 | 0.484 | 1 | 0.389 | up |
| Pyruvic acid | HMDB0000243 | C00022 | Glycine, serine and threonine metabolism | 33 | 0.499 | 1 | 0.398 | up |
| Pyruvic acid | HMDB0000243 | C00022 | Cysteine and methionine metabolism | 33 | 0.499 | 1 | 0.398 | up |
| Abbreviations: HMDB, Human Metabolome Database; KEGG, Kyoto Encyclopedia of Genes and Genomes. | | | | | | | | |

| **Table S7: Pathway enrichment analysis of targeted metabolomics (Normal Group vs. Disease Group)** | | | | | | | | |
| --- | --- | --- | --- | --- | --- | --- | --- | --- |
| Metabolism | HMDB | KEGG | Pathway | Total | Expected | Hits | Raw *P* | Sig |
| L-Asparagine | HMDB0000168 | C00152 | Aminoacyl-tRNA biosynthesis | 48 | 1.23 | 9 | 1.48E-06 | down |
| L-Histidine | HMDB0000177 | C00135 | Aminoacyl-tRNA biosynthesis | 48 | 1.23 | 9 | 1.48E-06 | down |
| L-Arginine | HMDB0000517 | C00062 | Aminoacyl-tRNA biosynthesis | 48 | 1.23 | 9 | 1.48E-06 | down |
| L-Glutamine | HMDB0000641 | C00064 | Aminoacyl-tRNA biosynthesis | 48 | 1.23 | 9 | 1.48E-06 | down |
| Glycine | HMDB0000123 | C00037 | Aminoacyl-tRNA biosynthesis | 48 | 1.23 | 9 | 1.48E-06 | down |
| L-Serine | HMDB0000187 | C00065 | Aminoacyl-tRNA biosynthesis | 48 | 1.23 | 9 | 1.48E-06 | down |
| L-Threonine | HMDB0000167 | C00188 | Aminoacyl-tRNA biosynthesis | 48 | 1.23 | 9 | 1.48E-06 | down |
| L-Tyrosine | HMDB0000158 | C00082 | Aminoacyl-tRNA biosynthesis | 48 | 1.23 | 9 | 1.48E-06 | down |
| L-Glutamic acid | HMDB0000148 | C00025 | Aminoacyl-tRNA biosynthesis | 48 | 1.23 | 9 | 1.48E-06 | up |
| L-Glutamic acid | HMDB0000148 | C00025 | Arginine biosynthesis | 14 | 0.359 | 4 | 0.000307 | up |
| L-Arginine | HMDB0000517 | C00062 | Arginine biosynthesis | 14 | 0.359 | 4 | 0.000307 | down |
| Citrulline | HMDB0000904 | C00327 | Arginine biosynthesis | 14 | 0.359 | 4 | 0.000307 | down |
| L-Glutamine | HMDB0000641 | C00064 | Arginine biosynthesis | 14 | 0.359 | 4 | 0.000307 | down |
| L-Glutamine | HMDB0000641 | C00064 | Purine metabolism | 64 | 1.64 | 7 | 0.000884 | down |
| Adenosine triphosphate | HMDB0000538 | C00002 | Purine metabolism | 64 | 1.64 | 7 | 0.000884 | up |
| ADP | HMDB0001341 | C00008 | Purine metabolism | 64 | 1.64 | 7 | 0.000884 | up |
| dGDP | HMDB0000960 | C00361 | Purine metabolism | 64 | 1.64 | 7 | 0.000884 | up |
| Inosine triphosphate | HMDB0000189 | C00081 | Purine metabolism | 64 | 1.64 | 7 | 0.000884 | up |
| dGTP | HMDB0001440 | C00286 | Purine metabolism | 64 | 1.64 | 7 | 0.000884 | up |
| Adenosine diphosphate ribose | HMDB0001178 | C00301 | Purine metabolism | 64 | 1.64 | 7 | 0.000884 | up |
| L-Serine | HMDB0000187 | C00065 | Glyoxylate and dicarboxylate metabolism | 32 | 0.82 | 5 | 0.00103 | down |
| Glycine | HMDB0000123 | C00037 | Glyoxylate and dicarboxylate metabolism | 32 | 0.82 | 5 | 0.00103 | down |
| L-Glutamic acid | HMDB0000148 | C00025 | Glyoxylate and dicarboxylate metabolism | 32 | 0.82 | 5 | 0.00103 | up |
| L-Glutamine | HMDB0000641 | C00064 | Glyoxylate and dicarboxylate metabolism | 32 | 0.82 | 5 | 0.00103 | down |
| 2-Phosphoglyceric acid | HMDB0000362 |  | Glyoxylate and dicarboxylate metabolism | 32 | 0.82 | 5 | 0.00103 | up |
| L-Serine | HMDB0000187 | C00065 | Glycine, serine and threonine metabolism | 33 | 0.846 | 5 | 0.00119 | down |
| Glycine | HMDB0000123 | C00037 | Glycine, serine and threonine metabolism | 33 | 0.846 | 5 | 0.00119 | down |
| 2-Phosphoglyceric acid | HMDB0000362 |  | Glycine, serine and threonine metabolism | 33 | 0.846 | 5 | 0.00119 | up |
| L-Threonine | HMDB0000167 | C00188 | Glycine, serine and threonine metabolism | 33 | 0.846 | 5 | 0.00119 | down |
| 3-Phosphoglyceric acid | HMDB0000807 | C00597 | Glycine, serine and threonine metabolism | 33 | 0.846 | 5 | 0.00119 | up |
| L-Glutamic acid | HMDB0000148 | C00025 | D-Glutamine and D-glutamate metabolism | 4 | 0.102 | 2 | 0.00372 | up |
| L-Glutamine | HMDB0000641 | C00064 | D-Glutamine and D-glutamate metabolism | 4 | 0.102 | 2 | 0.00372 | down |
| L-Glutamic acid | HMDB0000148 | C00025 | Nitrogen metabolism | 6 | 0.154 | 2 | 0.009 | up |
| L-Glutamine | HMDB0000641 | C00064 | Nitrogen metabolism | 6 | 0.154 | 2 | 0.009 | down |
| 4-Trimethylammoniobutanoic acid | HMDB0001161 | C01181 | Lysine degradation | 25 | 0.641 | 3 | 0.0242 | down |
| N6,N6,N6-Trimethyl-L-lysine | HMDB0001325 | C03793 | Lysine degradation | 25 | 0.641 | 3 | 0.0242 | down |
| L-Carnitine | HMDB0000062 | C00318 | Lysine degradation | 25 | 0.641 | 3 | 0.0242 | down |
| L-Glutamic acid | HMDB0000148 | C00025 | Alanine, aspartate and glutamate metabolism | 28 | 0.717 | 3 | 0.0327 | up |
| L-Glutamine | HMDB0000641 | C00064 | Alanine, aspartate and glutamate metabolism | 28 | 0.717 | 3 | 0.0327 | down |
| L-Asparagine | HMDB0000168 | C00152 | Alanine, aspartate and glutamate metabolism | 28 | 0.717 | 3 | 0.0327 | down |
| L-Serine | HMDB0000187 | C00065 | Cysteine and methionine metabolism | 33 | 0.846 | 3 | 0.0499 | down |
| L-Cystine | HMDB0000192 | C00491 | Cysteine and methionine metabolism | 33 | 0.846 | 3 | 0.0499 | up |
| 3-Phosphoglyceric acid | HMDB0000807 | C00597 | Cysteine and methionine metabolism | 33 | 0.846 | 3 | 0.0499 | up |
| Abbreviations: HMDB, Human Metabolome Database; KEGG, Kyoto Encyclopedia of Genes and Genomes. | | | | | | | | |

| **Table S8: Pathway enrichment analysis of targeted metabolomics (Non-DON Group vs. DON Group)** | | | | | | | | |
| --- | --- | --- | --- | --- | --- | --- | --- | --- |
| Metabolism | HMDB | KEGG | Pathway | Total | Expected | Hits | Raw *P* | Sig |
| Isocitric acid | HMDB0000193 | C00311 | Citrate cycle (TCA cycle) | 20 | 0.289 | 2 | 0.0324 | down |
| Citric acid | HMDB0000094 | C00158 | Citrate cycle (TCA cycle) | 20 | 0.289 | 2 | 0.0324 | up |
| Isocitric acid | HMDB0000193 | C00311 | Glyoxylate and dicarboxylate metabolism | 32 | 0.463 | 2 | 0.0762 | down |
| Citric acid | HMDB0000094 | C00158 | Glyoxylate and dicarboxylate metabolism | 32 | 0.463 | 2 | 0.0762 | up |
| Arachidonic acid | HMDB0001043 | C00219 | Biosynthesis of unsaturated fatty acids | 36 | 0.52 | 2 | 0.0935 | up |
| 8,11,14-Eicosatrienoic acid | HMDB0002925 | C03242 | Biosynthesis of unsaturated fatty acids | 36 | 0.52 | 2 | 0.0935 | up |
| Caffeine | HMDB0001847 | C07481 | Caffeine metabolism | 10 | 0.145 | 1 | 0.136 | down |
| Citrulline | HMDB0000904 | C00327 | Arginine biosynthesis | 14 | 0.202 | 1 | 0.185 | up |
| Inosine triphosphate | HMDB0000189 | C00081 | Purine metabolism | 64 | 0.925 | 2 | 0.236 | down |
| Deoxyadenosine | HMDB0000101 | C00559 | Purine metabolism | 64 | 0.925 | 2 | 0.236 | up |
| Aminoadipic acid | HMDB0000510 | C00956 | Lysine degradation | 25 | 0.361 | 1 | 0.307 | down |
| 3-Phosphoglyceric acid | HMDB0000807 | C00597 | Glycolysis / Gluconeogenesis | 26 | 0.376 | 1 | 0.317 | down |
| Citric acid | HMDB0000094 | C00158 | Alanine, aspartate and glutamate metabolism | 28 | 0.405 | 1 | 0.337 | up |
| 3-Phosphoglyceric acid | HMDB0000807 | C00597 | Glycine, serine and threonine metabolism | 33 | 0.477 | 1 | 0.385 | down |
| Abbreviations: HMDB, Human Metabolome Database; KEGG, Kyoto Encyclopedia of Genes and Genomes. | | | | | | | | |
